# Supplementary material for: Spearheading future omics analyses using dyngen, a multi-modal simulator of single cells
Source: Nat Commun. 2021 Jun 24;12:3942. doi: 10.1038/s41467-021-24152-2 (PMC8225657; doi:10.1038/s41467-021-24152-2)
Supplement: Supplementary file 1 — Supplementary Information [file 41467_2021_24152_MOESM1_ESM.pdf]

Supplementary Figures

Supplementary Table 1: **Feature comparison of single-cell synthetic data generators and simulators.** [1] As showcased by vignette. [2] As showcased by Van den Berge et al. [1].

	splatter	powsimR	PROSSTT	SymSim	dyngen
**Available modality outputs**					
- mRNA expression	✓	✓	✓	✓	✓
- Pre-mRNA expression					✓
- Protein expression					✓
- Promotor activity				✓	✓
- Reaction activity					✓
**Available ground-truth outputs**					
- True counts	✓			✓	✓
- Cluster labels	✓	✓		✓	✓
- Trajectory	✓		✓[1]	✓	✓
- Batch labels	✓				✓[1]
- Differential expression				✓	✓[2]
- Knocked down regulators					✓
- Regulatory network				✓	✓
- Cell-specific regulatory network					✓
**Emulate experimental effects**					
- Single-cell RNA sequencing	✓	✓	✓	✓	✓
- Batch effects	✓				✓[1]
- Knockdown experiment					✓
- Time-series					✓
- Snapshot					✓
**Evaluation applications**					
- Clustering	✓	✓		✓	
- Trajectory inference	✓		✓	✓	✓
- Network inference				✓	✓
- Cell-specific network inference					✓
- Differential expression	✓				
- Trajectory differential expression					✓[2]
- Batch effect correction	✓				✓
- RNA Velocity					✓
- Trajectory alignment					✓

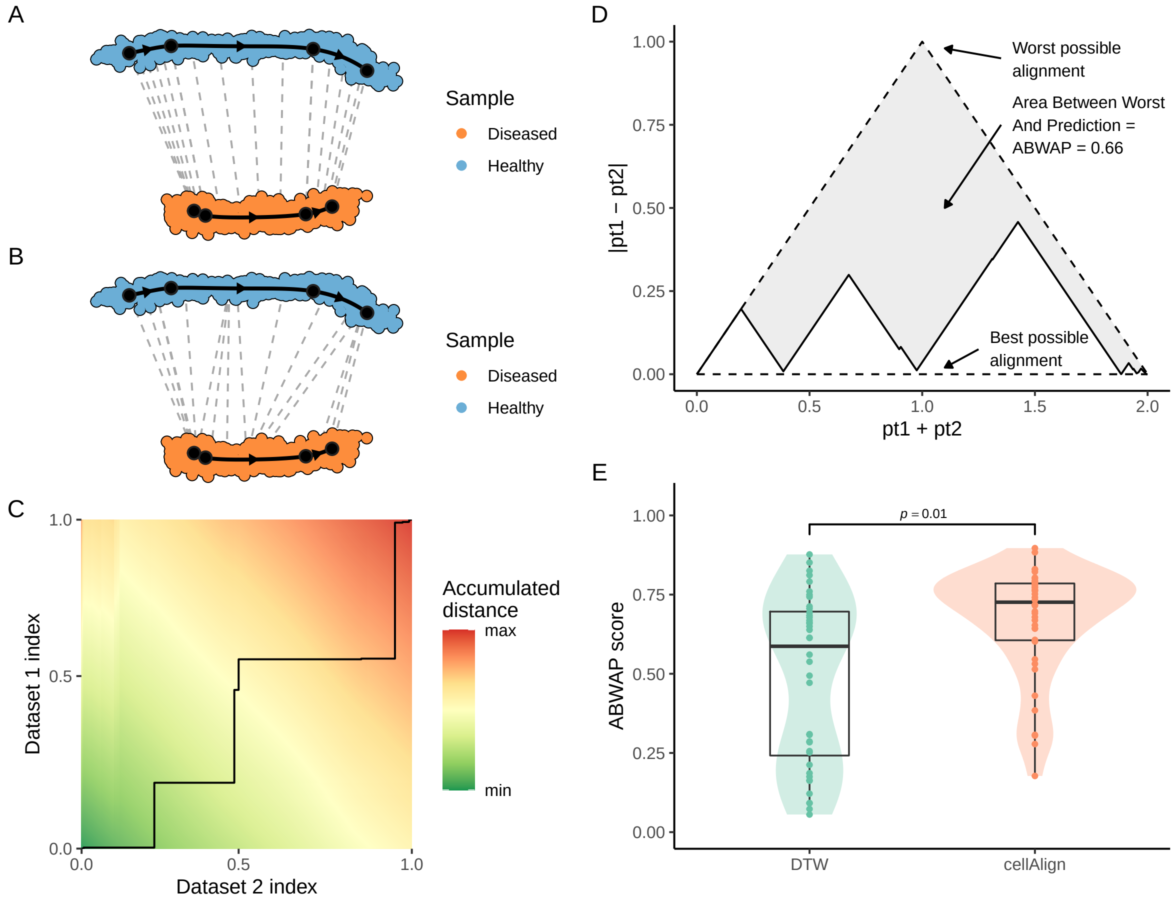

Supplementary Figure 1: **dyngen allows benchmarking of trajectory alignment methods.** **A**: An example linear dataset in need of trajectory alignment. Dashed lines represent the gold-standard alignment between the two trajectories according to the respective pseudotimes. **B**: Result of the DTW alignment on the two trajectories. **C**: DTW calculates an accumulated distance matrix. In this matrix, a warping path (shown in black), following a valley in the matrix from the bottom left to the top right corner is found. This shows how the trajectories best match each other. **D**: Illustration of the Area Between Worst and Prediction (ABWAP) metric. The warping path from subfigure C is mapped to the respective pseudotimes from both trajectories. The ABWAP score is equal to the area between the prediction and the worst possible prediction. **E:** An evaluation of DTW versus cellAlign on 40 different linear trajectories, in which cellAlign significantly outperforms DTW. Box plot denotes the Q_0 to Q_4 quartile values. Significance of differences between methods was tested with a non-parametric paired two-sided Durbin-Conover test. Test statistics and p-values for all combinations are reported in the Source Data file.

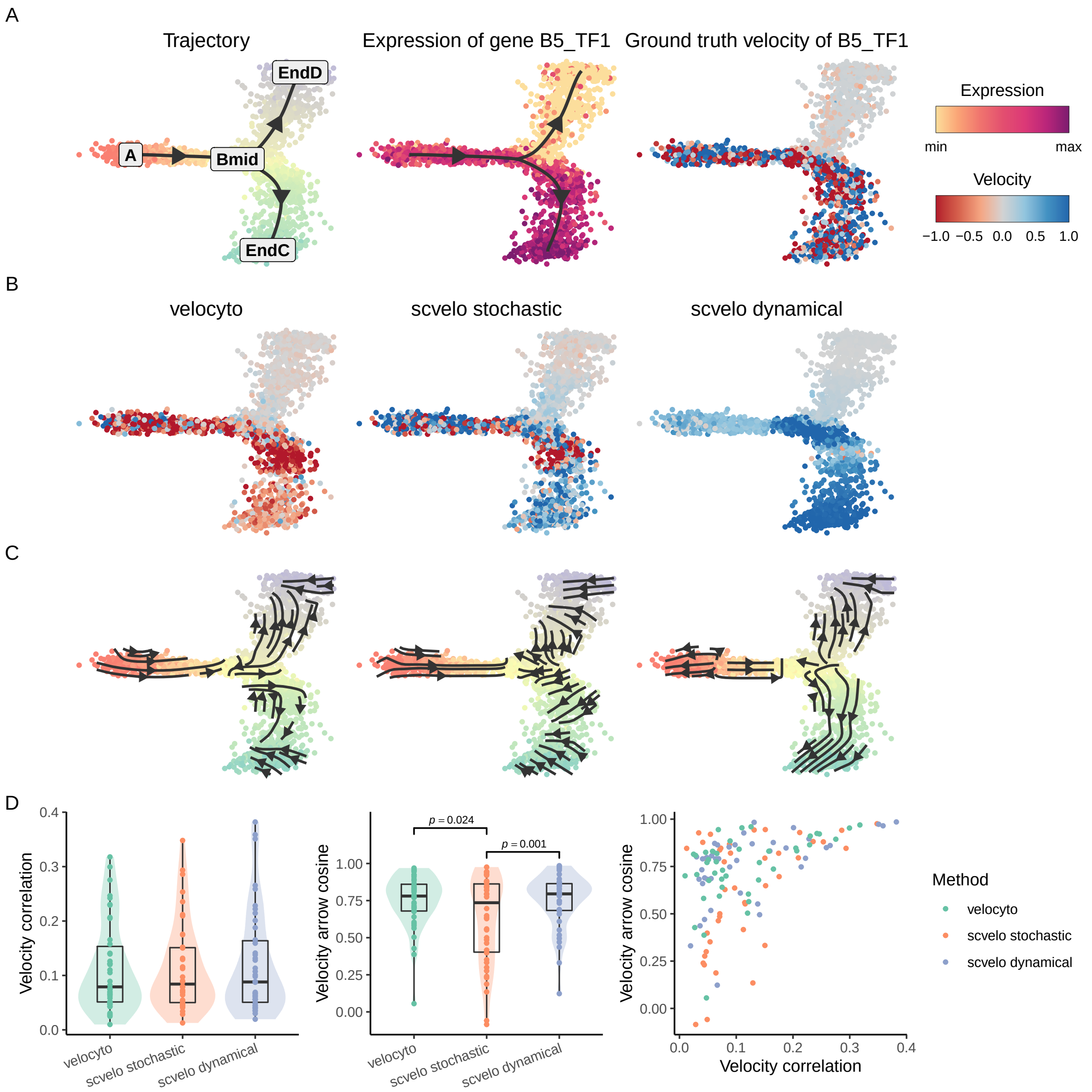

Supplementary Figure 2: **dyngen allows benchmarking of RNA velocity methods.** **A:** The ground-truth information of a bifurcating dataset: ground-truth trajectory (left), gene expression of a gene B5_TF1 (middle), and the RNA velocity of B5_TF1 (right). **B:** The RNA velocity estimates of gene B5_TF1 by the different methods. **C:** The velocity stream plots produced from the predictions of each method, as generated by scvelo. **D:** The predictions scored by two different metrics, the velocity correlation and the velocity arrow cosine. The velocity correlation is the correlation between the ground-truth velocity (A, right) and the predicted velocity (B). The velocity arrow cosine is the cosine similarity between the direction of segments of the ground-truth trajectory (A, left) and the RNA velocity values calculated at those points (C). Box plots denote the Q_0 to Q_4 quartile values. Significance of differences between methods was tested with pairwise non-parametric paired two-sided Durbin-Conover tests, Holm corrected for multiple testing. Test statistics and p-values for all combinations are reported in the Source Data file.

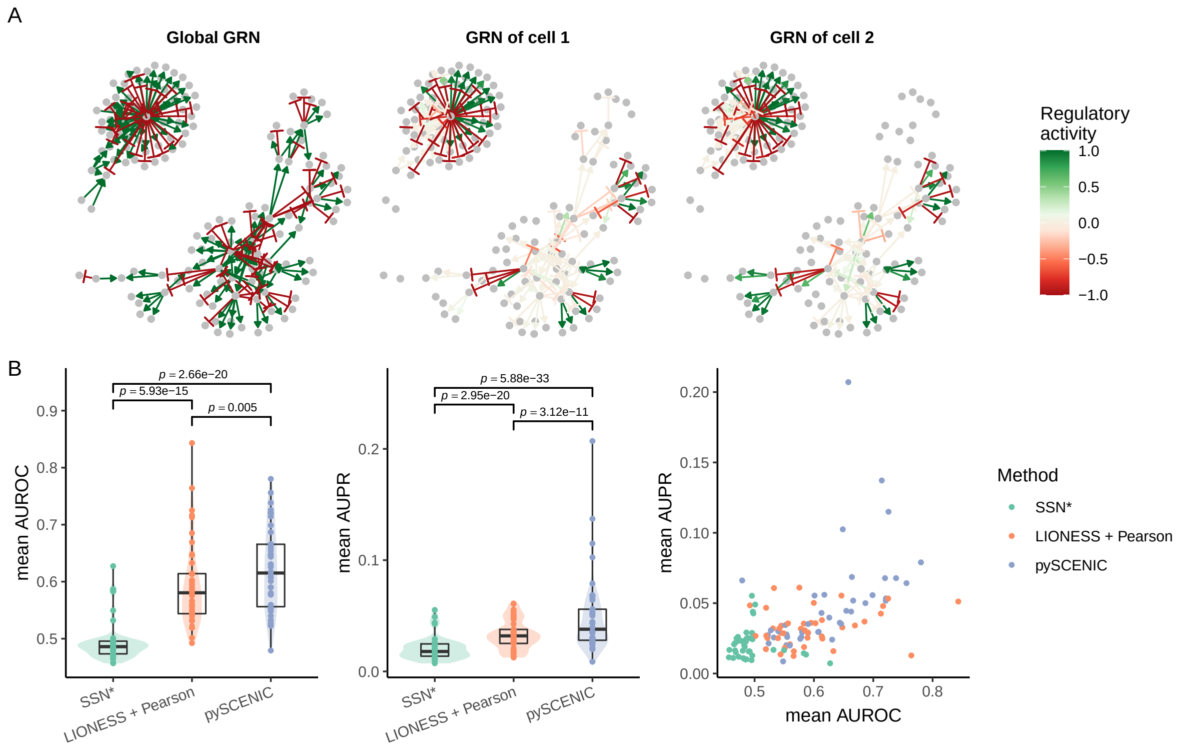

Supplementary Figure 3: **dyngen allows benchmarking Cell-specific Network Inference (CSNI) methods. A:** A cell is simulated using the global gene regulatory network (GRN, top left). However, at any particular state in the simulation, only a fraction of the gene regulatory interactions are active. **B:** CSNI methods were executed to predict the regulatory interactions that are active in each cell specifically. Using the ground-truth cell-specific GRN, the performance of each method was quantified on 42 dyngen datasets. Box plots denote the Q_0 to Q_4 quartile values. Significance of differences between methods was tested with pairwise non-parametric paired two-sided Durbin-Conover tests, Holm corrected for multiple testing. Test statistics and p-values for all combinations are reported in the Source Data file.

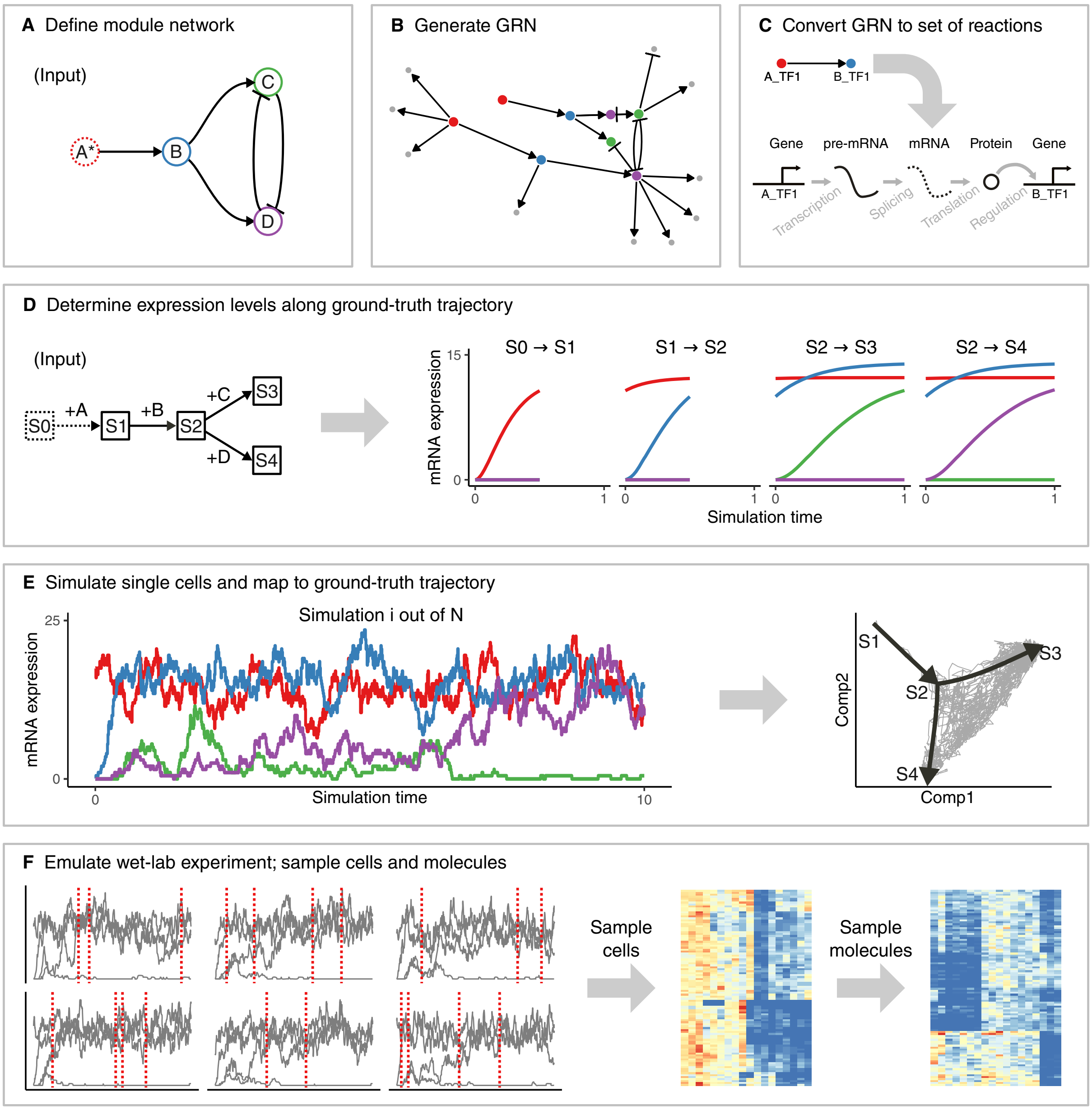

Supplementary Figure 4: **The workflow of dyngen consists of six main steps. A:** The user needs to specify the desired module network or use a predefined module network. The module network is what determines the dynamic behaviour of simulated cells. **B:** The number of desired transcription factors (which drive the desired dynamic process) are amongst the given modules and adds regulatory interactions according to the module network. Additional target genes (which do not influence the dynamic process) are added by sampling interactions from GRN interaction databases. **C:** Each gene regulatory interaction in the GRN is converted to a set of biochemical reactions. **D:** Along with the module network, the user also needs to specify the backbone structure of expected cell states. The average expression of each edge in the backbone is simulated by activating a restricted set of genes for each edge. **E:** Multiple Gillespie SSA simulations are run using the reactions defined in step C. The counts of each of the molecules at each time step are extracted. Each time step is mapped to a point in the backbone. **F:** The molecule levels of multiple simulations are shown over time (left). From each simulation, multiple cells are sampled (from left to middle). Technical noise from profiling is simulated by sampling molecules from the set of molecules inside each cell (from middle to right).

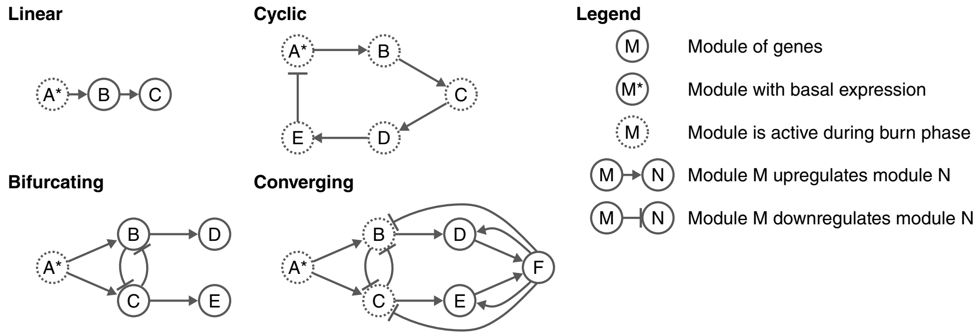

Supplementary Figure 5: **The module network determines the type of dynamic process which simulated cells will undergo.** A module network describes the regulatory interactions between sets of transcription factors which drive the desired dynamic process.

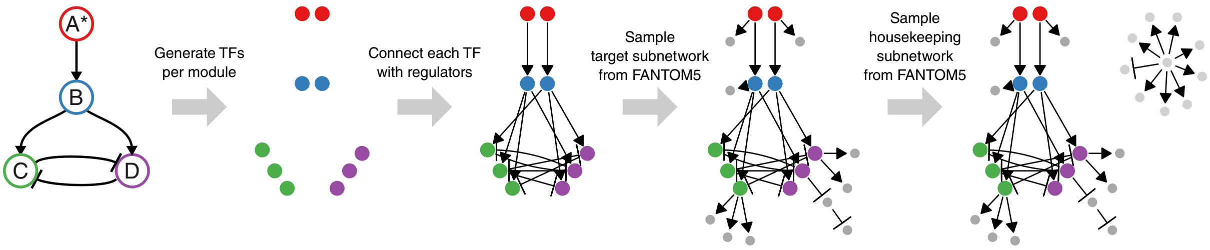

Supplementary Figure 6: **Generating the feature network from a backbone consists of four main steps.**

Supplementary Table 2: **Reactions affecting the abundance levels of pre-mRNA x_G, mature mRNA y_G and proteins z_G of gene G.** Define the set of regulators of G as R_G, the set of upregulating regulators of G as R_G^+, and the set of down-regulating regulators of G as R_G^-. Parameters used in the propensity formulae are defined in Supplementary Table 3.

Reaction	Effect	Propensity
Transcription	$\emptyset \rightarrow \mathrm{x}_G$	$\mathrm{xpr}_G \times \frac{\mathrm{bas}_G - \mathrm{ind}_G^{\lvert \mathrm{R}_G^+ \rvert} + \prod_{H \in \mathrm{R}_G^+} (\mathrm{ind}_G + \mathrm{bind}_{G,H})}{\prod_{H \in \mathrm{R}_G} (1 + \mathrm{bind}_{G,H})}$
Splicing	$\mathrm{x}_G \rightarrow \mathrm{y}_G$	$\mathrm{ysr}_G \times \mathrm{x}_G$
Translation	$\mathrm{y}_G \rightarrow \mathrm{y}_G + \mathrm{z}_G$	$\mathrm{zpr}_G \times \mathrm{y}_G$
Pre-mRNA degradation	$\mathrm{x}_G \rightarrow \emptyset$	$\mathrm{ydr}_G \times \mathrm{x}_G$
Mature mRNA degradation	$\mathrm{y}_G \rightarrow \emptyset$	$\mathrm{ydr}_G \times \mathrm{y}_G$
Protein degradation	$\mathrm{z}_G \rightarrow \emptyset$	$\mathrm{zdr}_G \times \mathrm{z}_G$

Supplementary Table 3: **Default parameters defined for the calculation of reaction propensity functions.**

Parameter	Symbol	Definition
Transcription rate	xpr_G	$\in U(10, 20)$
Splicing rate	ysr_G	$= \ln(2)\ /\ 2$
Translation rate	zpr_G	$\in U(100, 150)$
(Pre-)mRNA half-life	yhl_G	$\in U(2.5, 5)$
Protein half-life	zhl_G	$\in U(5, 10)$
Interaction strength	$\text{str}_{G,H}$	$\in 10^{U(0,2)}$ *
Hill coefficient	$\text{hill}_{G,H}$	$\in U(0.5, 2)$ *
Independence factor	ind_G	$\in U(0, 1)$ *
(Pre-)mRNA degradation rate	ydr_G	$= \ln(2)\ /\ \text{yhl}_G$
Protein degradation rate	zdr_G	$= \ln(2)\ /\ \text{zhl}_G$
Dissociation constant	dis_H	$= 0.5 \times \frac{\text{xpr}_H \times \text{ysr}_H \times \text{zpr}_H}{(\text{ydr}_H + \text{ysr}_H) \times \text{ydr}_H \times \text{zdr}_H}$
Binding strength	$\text{bind}_{G,H}$	$= \text{str}_{G,H} \times (\text{z}_H\ /\ \text{dis}_H)^{\text{hill}_{G,H}}$
Basal expression	bas_G	$= \begin{cases} 1 & \text{if } \text{R}^+_G = \emptyset \\ 0.0001 & \text{if } \text{R}^-_G = \emptyset \text{ and } \text{R}^+_G \neq \emptyset \text{ *} \\ 0.5 & \text{otherwise} \end{cases}$

*: unless G is a TF, then the value is determined by the backbone.

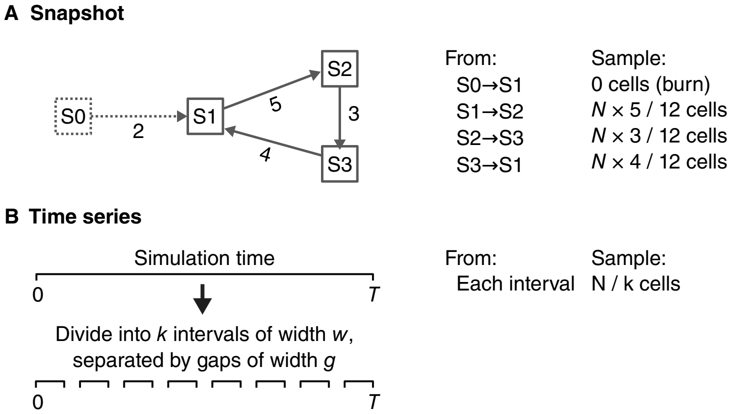

Supplementary Figure 7: **Two approaches can be used to sample cells from simulations: snapshot and time-series.**

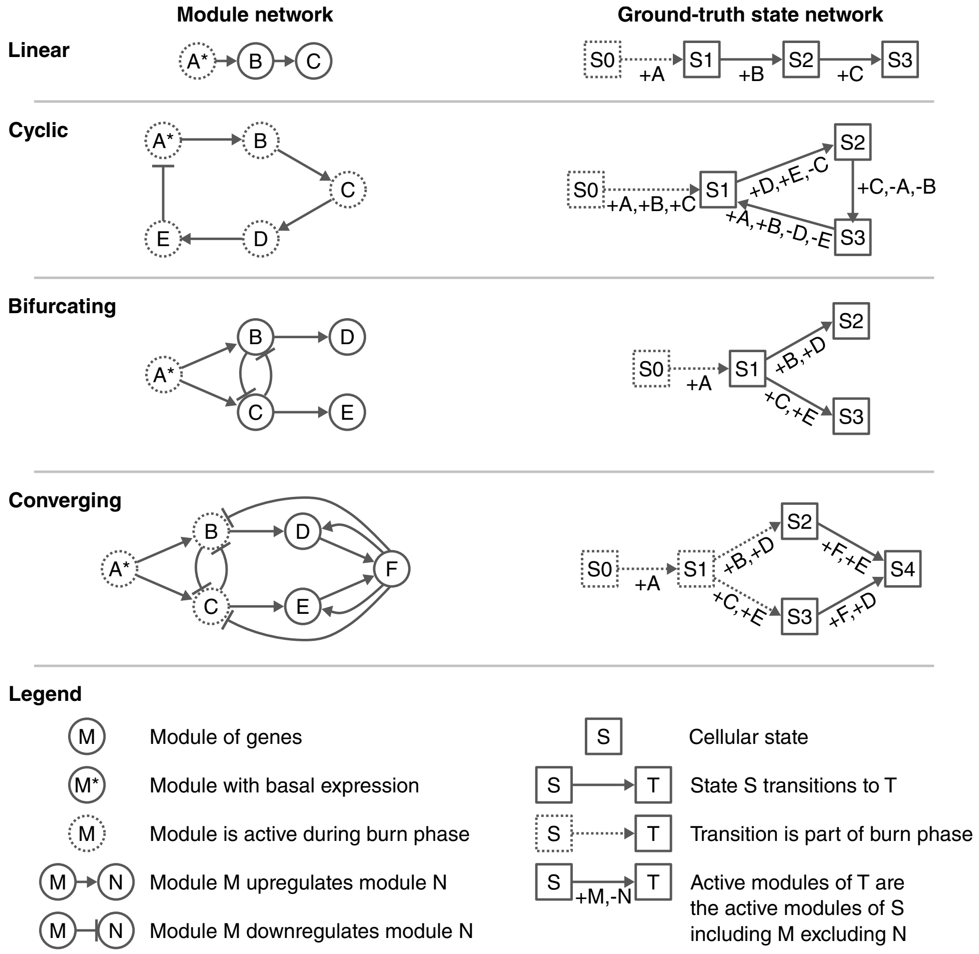

Supplementary Figure 8: **Examples of the ground-truth state networks which need to be provided alongside the module network.**

Supplementary Note 1: Comparison to reference dataset

In this vignette, we will take a look at characteristic features of dyngen versus the reference dataset it uses. To this end, we'll be using `countsimQC` [2] to calculate key statistics of both datasets and create comparative visualisations.

Run dyngen simulation

We use an internal function from the dyngen package to download and cache one of the reference datasets.

```
library(tidyverse)
library(dyngen)

set.seed(1)

data("realcounts", package = "dyngen")
name_realcounts <- paste0(
  "zenodo_1443566_real_silver_",
  "bone-marrow-mesenchyme-erythrocyte-differentiation_mca"
)
url_realcounts <- realcounts %>% filter(name == name_realcounts) %>% pull(url)
cache_dir <- getOption("dyngen_download_cache_dir")
realcount <-
  dyngen:::.download_cacheable_file(url_realcounts, cache_dir, verbose = FALSE)
```

We run a simple dyngen dataset as follows, where the number of cells and genes are determined by the size of the reference dataset.

```
backbone <- backbone_bifurcating_loop()

num_cells <- nrow(realcount)
num_feats <- ncol(realcount)
num_tfs <- nrow(backbone$module_info)
num_tar <- round((num_feats - num_tfs) / 2)
num_hks <- num_feats - num_tfs - num_tar

config <-
  initialise_model(
    backbone = backbone,
    num_cells = num_cells,
    num_tfs = num_tfs,
    num_targets = num_tar,
    num_hks = num_hks,
    gold_standard_params = gold_standard_default(),
    simulation_params = simulation_default(
      total_time = 1000,
      experiment_params = simulation_type_wild_type(num_simulations = 100)
    ),
    experiment_params = experiment_snapshot(
      realcount = realcount
    ),
    verbose = FALSE
  )
```

```
# the simulation is being sped up because rendering all vignettes with one core

# for pkgdown can otherwise take a very long time
set.seed(1)
```

```r
config <-
  initialise_model(
    backbone = backbone,
    num_cells = num_cells,
    num_tfs = num_tfs,
    num_targets = num_tar,
    num_hks = num_hks,
    verbose = interactive(),
    download_cache_dir = tools::R_user_dir("dyngen", "data"),
    simulation_params = simulation_default(
      total_time = 1000,
      census_interval = 2,
      ssa_algorithm = ssa_etl(tau = 300/3600),
      experiment_params = simulation_type_wild_type(num_simulations = 10)
    ),
    experiment_params = experiment_snapshot(
      realcount = realcount
    )
  )
```

```r
out <- generate_dataset(config, make_plots = TRUE)
```

```
## Generating TF network
## Sampling feature network from real network
## Generating kinetics for 3025 features
## Generating formulae
## Generating gold standard mod changes
## Precompiling reactions for gold standard
## Running gold simulations
##   |                                                  | 0 % elapsed=00s     |========
## Precompiling reactions for simulations
## Running 10 simulations
## Mapping simulations to gold standard
## Performing dimred
## Simulating experiment
## Wrapping dataset
## Making plots
```

```r
out$plot
```

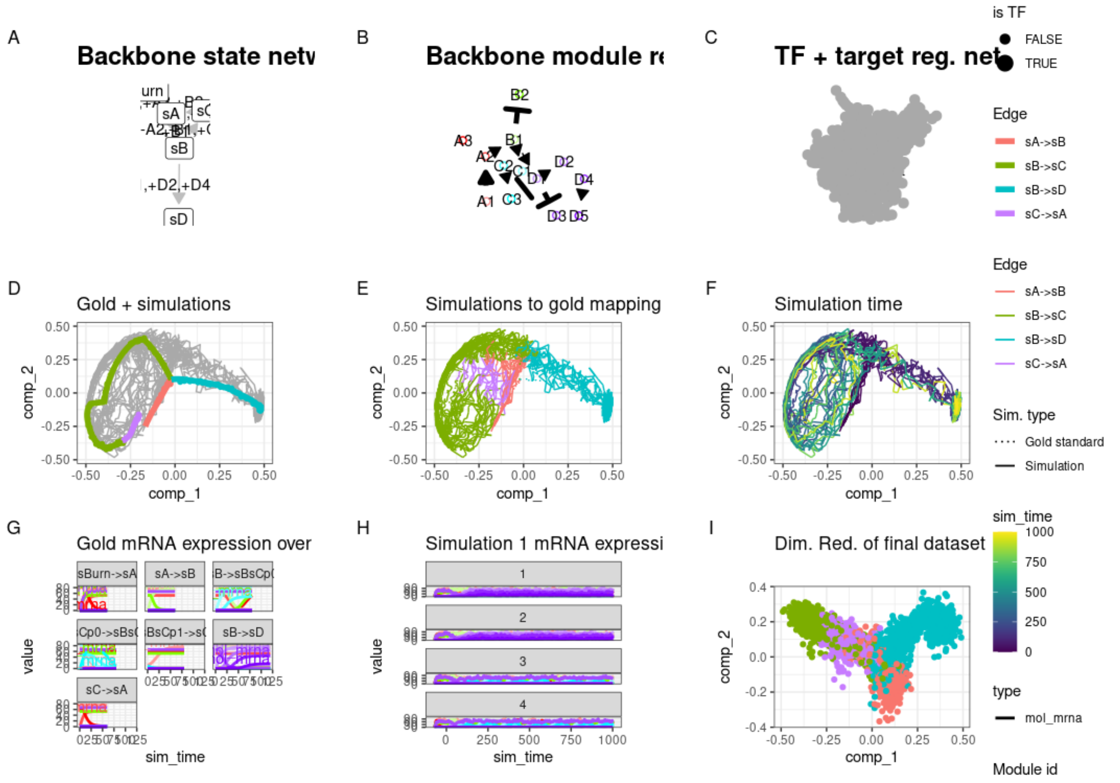

Both datasets are stored in a list for easy usage by countsimQC.

```
datasets <- list(
  real = t(as.matrix(realcount)),
  dyngen = t(as.matrix(out$dataset$counts))
)

ddsList <- lapply(datasets, function(ds) {
  DESeq2::DESeqDataSetFromMatrix(
    countData = round(as.matrix(ds)),
    colData = data.frame(sample = seq_len(ncol(ds))),
    design = ~1
  )
})
```

Run countsimQC computations

Below are some computations countsimQC makes. Normally these are not visible to the user, but for the sake of transparency these are included in the vignette.

```
library(countsimQC)


### Define helper objects
nDatasets <- length(ddsList)
colRow <- c(2, 1)
panelSize <- 4
thm <-
  theme_bw() +
  theme(
    axis.text = element_text(size = 15),
    axis.title = element_text(size = 14),
    strip.text = element_text(size = 15)
```

```
  )
```

Compute key characteristics

```
obj <- countsimQC:::calculateDispersionsddsList(ddsList = ddsList, maxNForDisp = Inf)

sampleCorrDF <- countsimQC:::calculateSampleCorrs(ddsList = obj, maxNForCorr = 500)

featureCorrDF <- countsimQC:::calculateFeatureCorrs(ddsList = obj, maxNForCorr = 500)
```

Summarize sample characteristics

```
sampleDF <- map2_df(obj, names(obj), function(x, dataset_name) {
  tibble(
    dataset = dataset_name,
    Libsize = colSums(x$dge$counts),
    Fraczero = colMeans(x$dge$counts == 0),
    TMM = x$dge$samples$norm.factors,
    EffLibsize = Libsize * TMM
  )
})
```

Summarize feature characteristics

```
featureDF <- map2_df(obj, names(obj), function(x, dataset_name) {
  rd <- SummarizedExperiment::rowData(x$dds)
  tibble(
    dataset = dataset_name,
    Tagwise = sqrt(x$dge$tagwise.dispersion),
    Common = sqrt(x$dge$common.dispersion),
    Trend = sqrt(x$dge$trended.dispersion),
    AveLogCPM = x$dge$AveLogCPM,
    AveLogCPMDisp = x$dge$AveLogCPMDisp,
    average_log2_cpm = apply(edgeR::cpm(x$dge, prior.count = 2, log = TRUE), 1, mean),
    variance_log2_cpm = apply(edgeR::cpm(x$dge, prior.count = 2, log = TRUE), 1, var),
    Fraczero = rowMeans(x$dge$counts == 0),
    dispGeneEst = rd$dispGeneEst,
    dispFit = rd$dispFit,
    dispFinal = rd$dispersion,
    baseMeanDisp = rd$baseMeanDisp,
    baseMean = rd$baseMean
  )
})
```

Summarize data set characteristics

```
datasetDF <- map2_df(obj, names(obj), function(x, dataset_name) {
  tibble(
    dataset = dataset_name,
    prior_df = paste0("prior.df = ", round(x$dge$prior.df, 2)),
    nVars = nrow(x$dge$counts),
    nSamples = ncol(x$dge$counts),
    AveLogCPMDisp = 0.8 * max(featureDF$AveLogCPMDisp),
    Tagwise = 0.9 * max(featureDF$Tagwise)
  )
})
```

Data set dimensions

These bar plots show the number of samples (columns) and features (rows) in each data set.

Number of samples (columns)

```
ggplot(datasetDF, aes(x = dataset, y = nSamples, fill = dataset)) +
  geom_bar(stat = "identity", alpha = 0.5) +
  xlab("") + ylab("Number of samples (columns)") +
  thm + theme(axis.text.x = element_text(angle = 90, hjust = 1, vjust = 0.5))
```

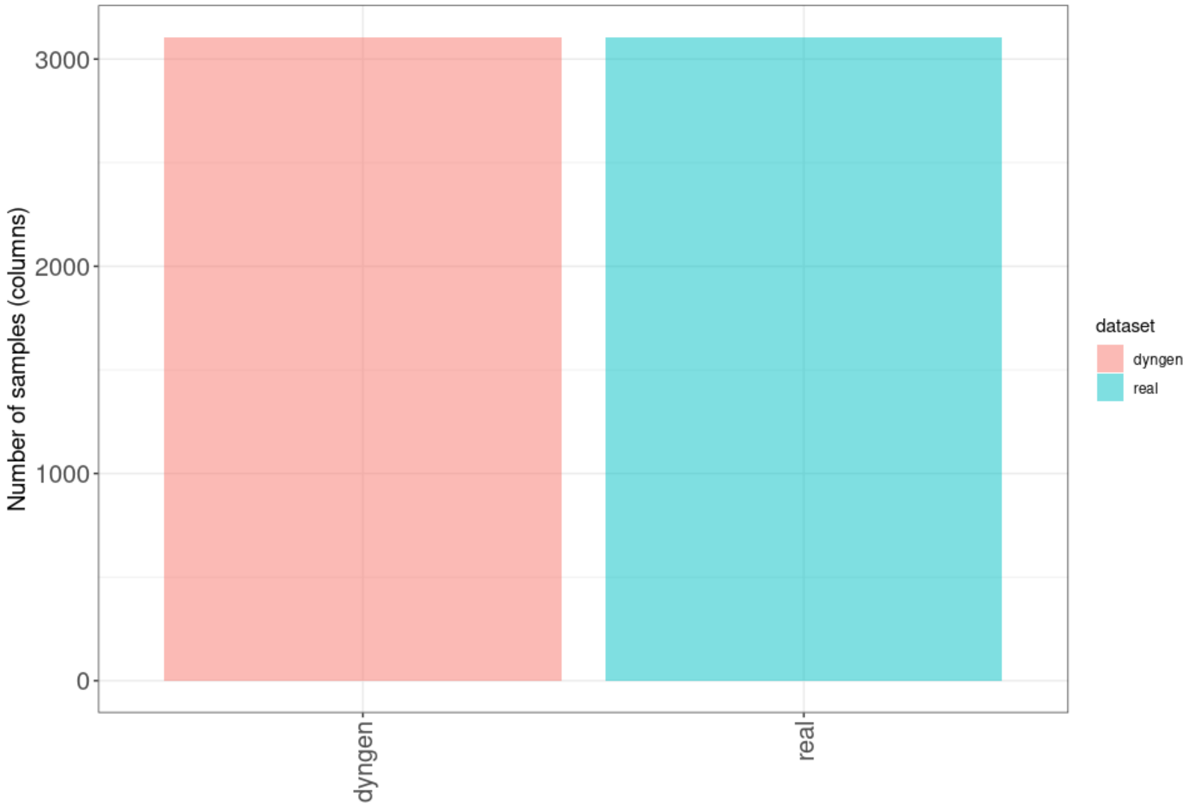

Number of features (rows)

```
ggplot(datasetDF, aes(x = dataset, y = nVars, fill = dataset)) +
  geom_bar(stat = "identity", alpha = 0.5) +
  xlab("") + ylab("Number of features (rows)") +
  thm + theme(axis.text.x = element_text(angle = 90, hjust = 1, vjust = 0.5))
```

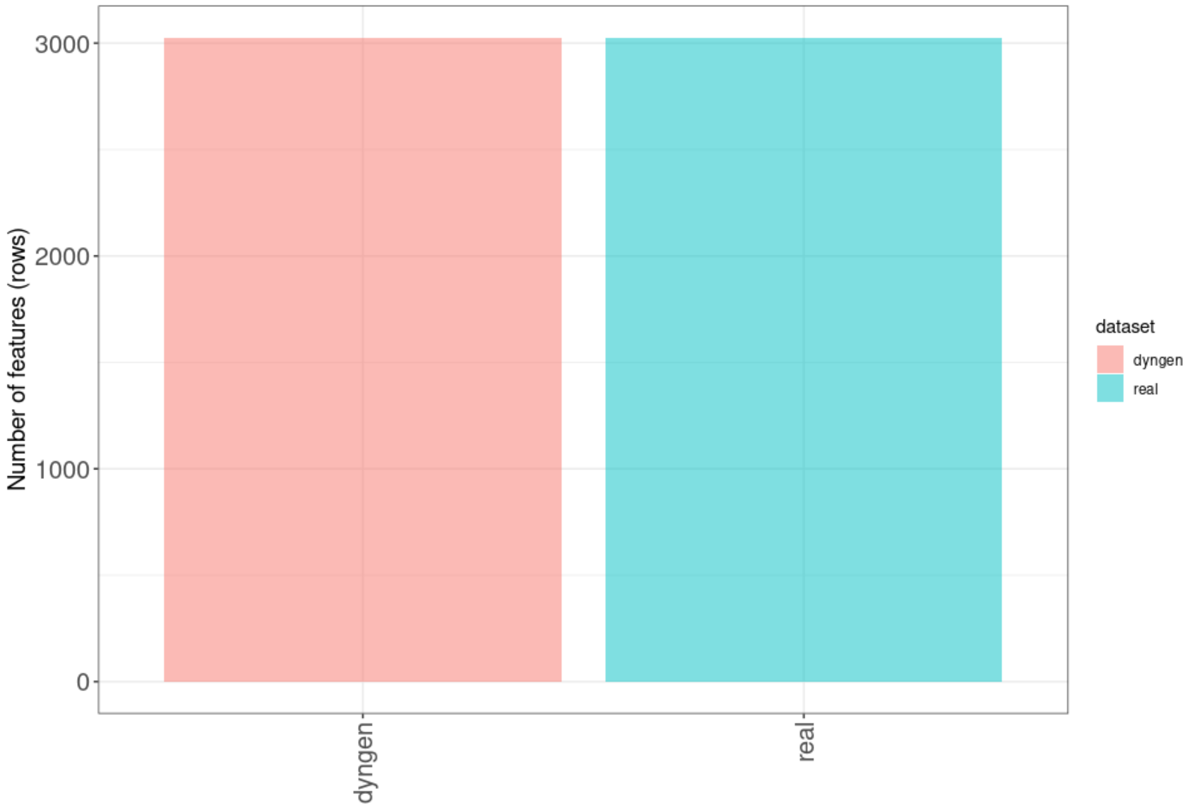

Dispersion/BCV plots

Disperson/BCV plots show the association between the average abundance and the dispersion or "biological coefficient of variation" (sqrt(dispersion)), as calculated by `edgeR` [3] and `DESeq2` [4]. In the `edgeR` plot, the estimate of the prior degrees of freedom is indicated.

edgeR The black dots represent the tagwise dispersion estimates, the red line the common dispersion and the blue curve represents the trended dispersion estimates. For further information about the dispersion estimation in `edgeR`, see Chen et al. [5].

```
ggplot(featureDF %>% dplyr::arrange(AveLogCPMDisp),
       aes(x = AveLogCPMDisp, y = Tagwise)) +
  geom_point(size = 0.25, alpha = 0.5) +
  facet_wrap(~dataset, nrow = colRow[2]) +
  geom_line(aes(y = Trend), color = "blue", size = 1.5) +
  geom_line(aes(y = Common), color = "red", size = 1.5) +
  geom_text(data = datasetDF, aes(label = prior_df)) +
  xlab("Average log CPM") + ylab("Biological coefficient of variation") +
  thm
```

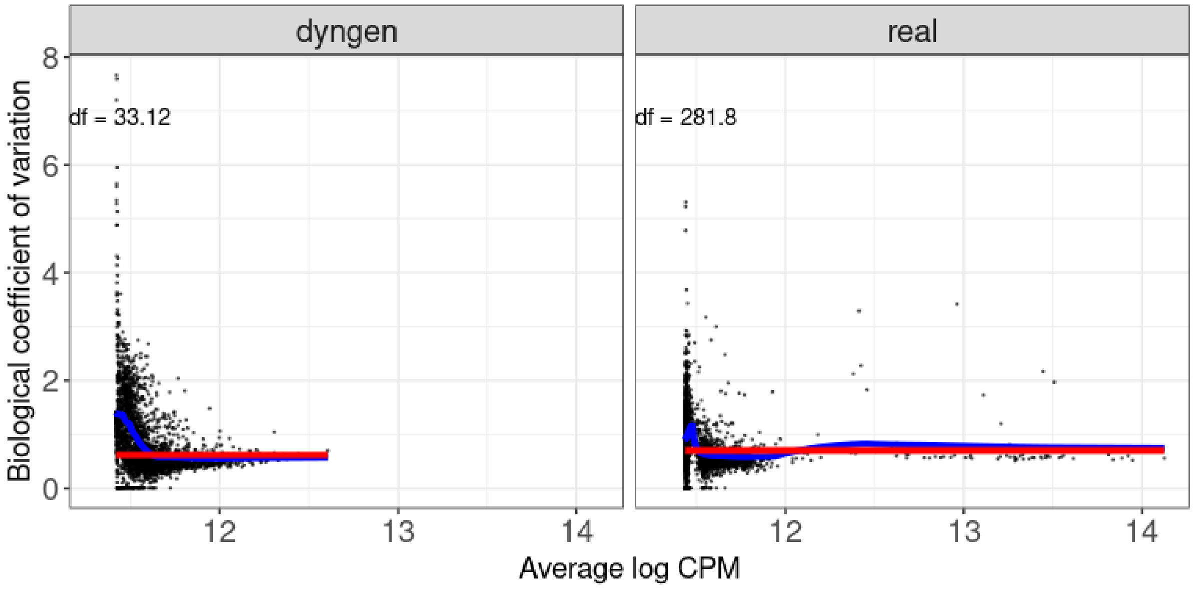

DESeq2

The black dots are the gene-wise dispersion estimates, the red curve the fitted mean-dispersion relationship and the blue circles represent the final dispersion estimates.For further information about the dispersion estimation in DESeq2, see Love et al. [4].

```
ggplot(featureDF %>% dplyr::arrange(baseMeanDisp),
       aes(x = baseMeanDisp, y = dispGeneEst)) +
  geom_point(size = 0.25, alpha = 0.5) +
  facet_wrap(~dataset, nrow = colRow[2]) + scale_x_log10() + scale_y_log10() +
  geom_point(aes(y = dispFinal), color = "lightblue", shape = 21) +
  geom_line(aes(y = dispFit), color = "red", size = 1.5) +
  xlab("Base mean") + ylab("Dispersion") +
  thm
```

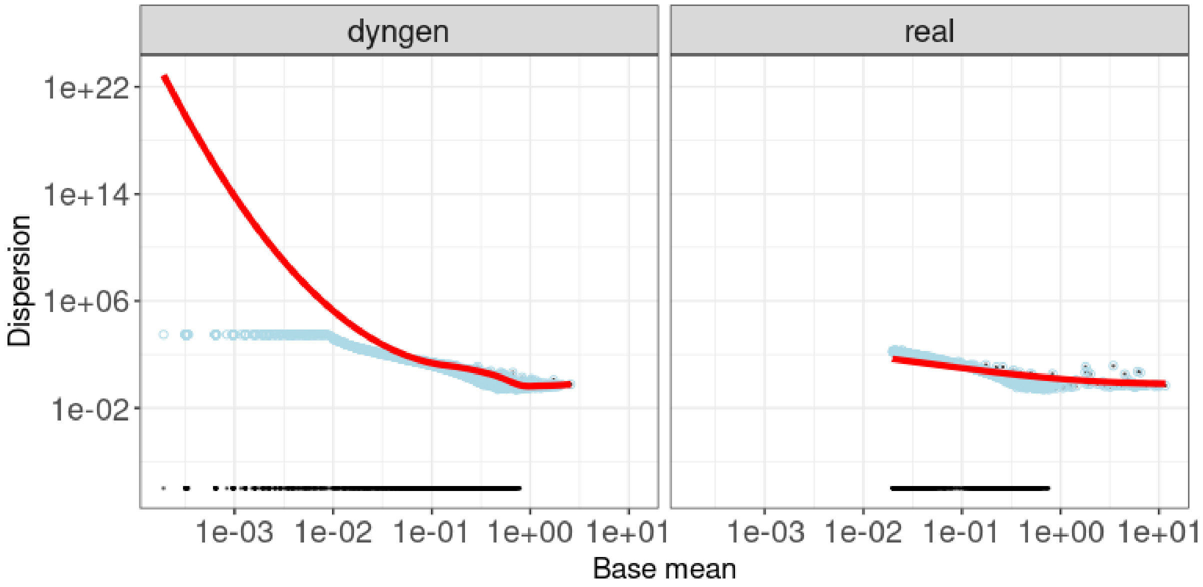

Mean-variance plots

This scatter plot shows the relation between the empirical mean and variance of the features. The difference between these mean-variance plots and the mean-dispersion plots above is that the plots

in this section do not take the information about the experimental design and sample grouping into account, but simply display the mean and variance of log2(CPM) estimates across all samples, calculated using the `cpm` function from `edgeR` [3], with a prior count of 2.

```
ggplot(featureDF, aes(x = average_log2_cpm, y = variance_log2_cpm)) +
  geom_point(size = 0.75, alpha = 0.5) +
  facet_wrap(~dataset, nrow = colRow[2]) +
  xlab("Mean of log2(CPM)") + ylab("Variance of log2(CPM)") +
  thm
```

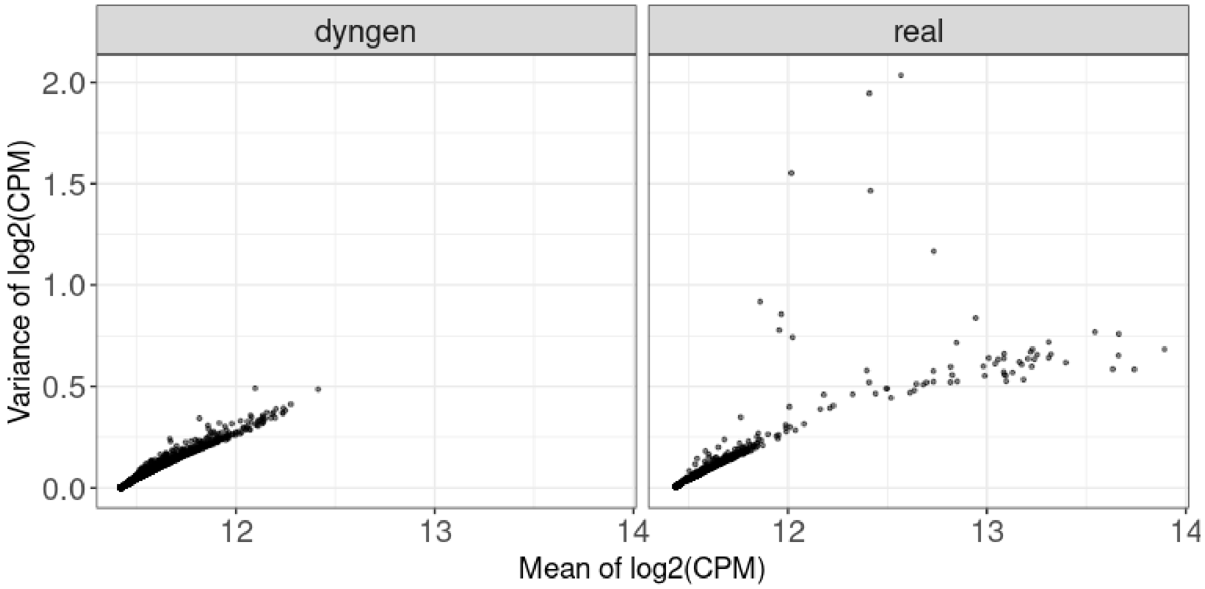

Library sizes

This plot shows a histogram of the total read count per sample, i.e., the column sums of the respective data matrices.

```
ggplot(sampleDF, aes(x = Libsize)) + geom_histogram(bins = 30) +
  facet_wrap(~dataset, nrow = colRow[2]) +
  xlab("Library size") + thm
```

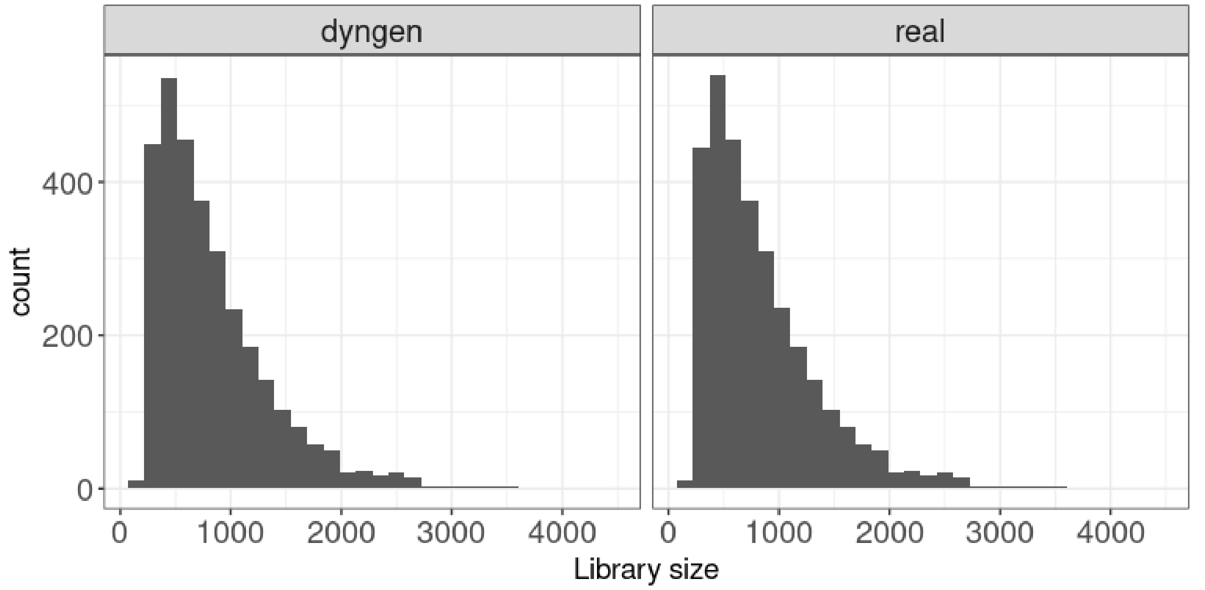

TMM normalization factors

This plot shows a histogram of the TMM normalization factors [6], intended to adjust for differences in RNA composition, as calculated by `edgeR` [3].

```
ggplot(sampleDF, aes(x = TMM)) + geom_histogram(bins = 30) +
  facet_wrap(~dataset, nrow = colRow[2]) +
  xlab("TMM normalization factor") + thm
```

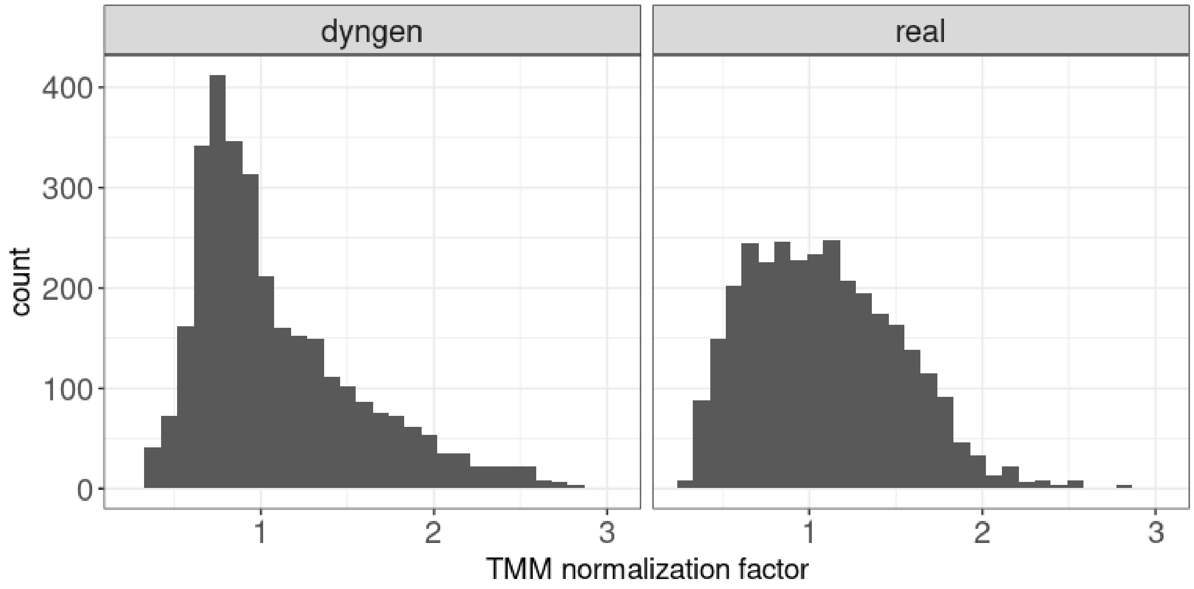

Effective library sizes

This plot shows a histogram of the "effective library sizes," defined as the total count per sample multiplied by the corresponding TMM normalization factor.

```
ggplot(sampleDF, aes(x = EffLibsize)) + geom_histogram(bins = 30) +
  facet_wrap(~dataset, nrow = colRow[2]) +
  xlab("Effective library size") + thm
```

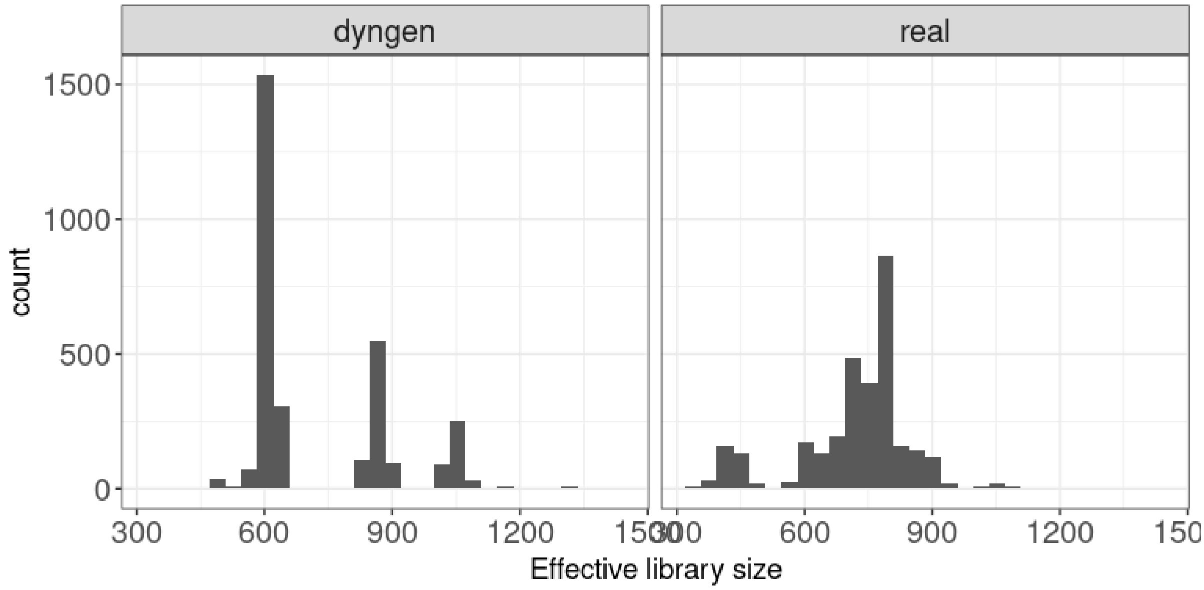

Expression distributions (average log CPM)

This plot shows the distribution of average abundance values for the features. The abundances are log CPM values calculated by `edgeR`.

```
ggplot(featureDF, aes(x = AveLogCPM)) + geom_histogram(bins = 30) +
  facet_wrap(~dataset, nrow = colRow[2]) +
  xlab("Average log CPM") + thm
```

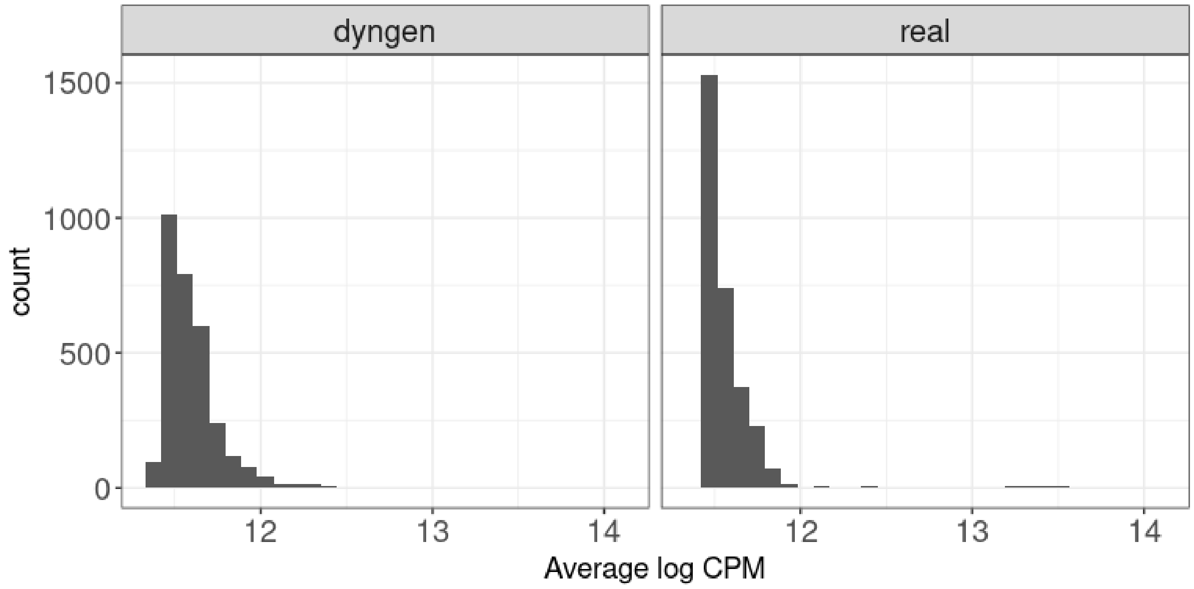

Fraction zeros per sample

This plot shows the distribution of the fraction of zeros observed per sample (column) in the count matrices.

```
ggplot(sampleDF, aes(x = Fraczero)) + geom_histogram(bins = 30) +
  facet_wrap(~dataset, nrow = colRow[2]) +
  xlab("Fraction zeros per sample") + thm
```

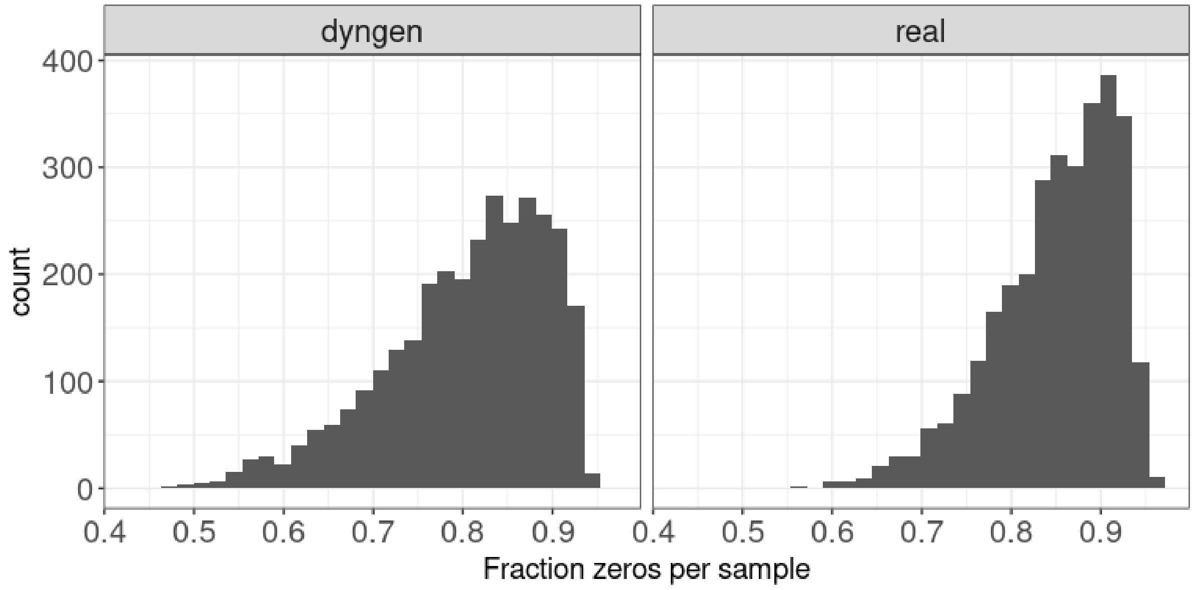

Fraction zeros per feature

This plot illustrates the distribution of the fraction of zeros observed per feature (row) in the count matrices.

```
ggplot(featureDF, aes(x = Fraczero)) + geom_histogram(bins = 30) +
  facet_wrap(~dataset, nrow = colRow[2]) +
  xlab("Fraction zeros per feature") + thm
```

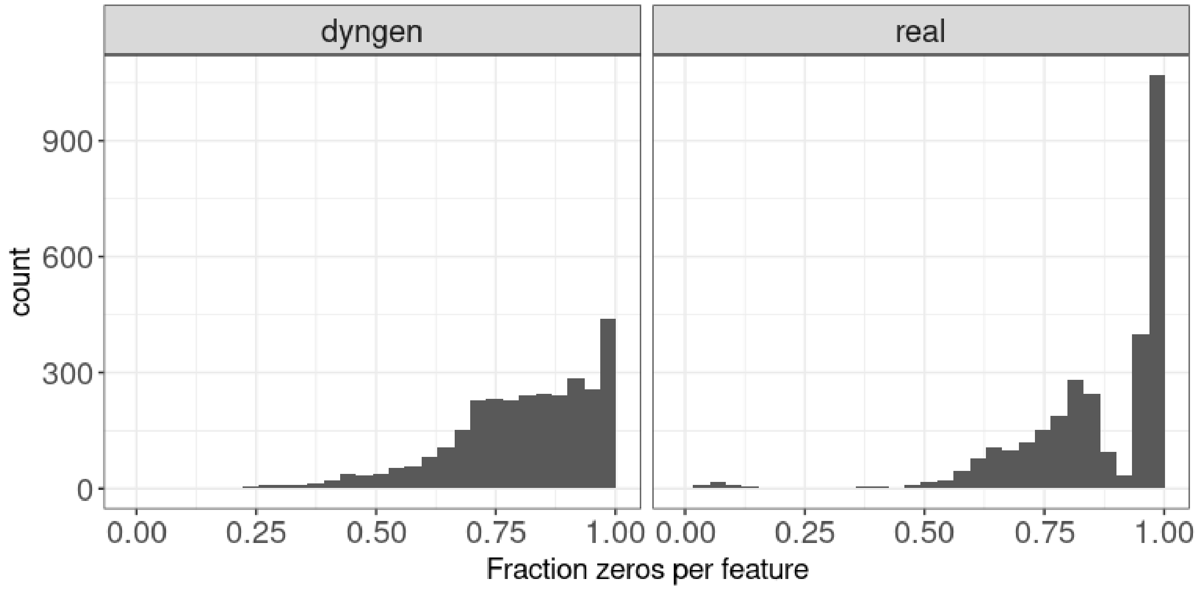

Sample-sample correlations

The plot below shows the distribution of Spearman correlation coefficients for pairs of samples, calculated from the log(CPM) values obtained via the `cpm` function from `edgeR`, with a prior.count of 2.

```
ggplot(sampleCorrDF, aes(x = Correlation)) + geom_histogram(bins = 30) +
  facet_wrap(~dataset, nrow = colRow[2]) +
  xlab("Sample-sample correlation") + thm
```

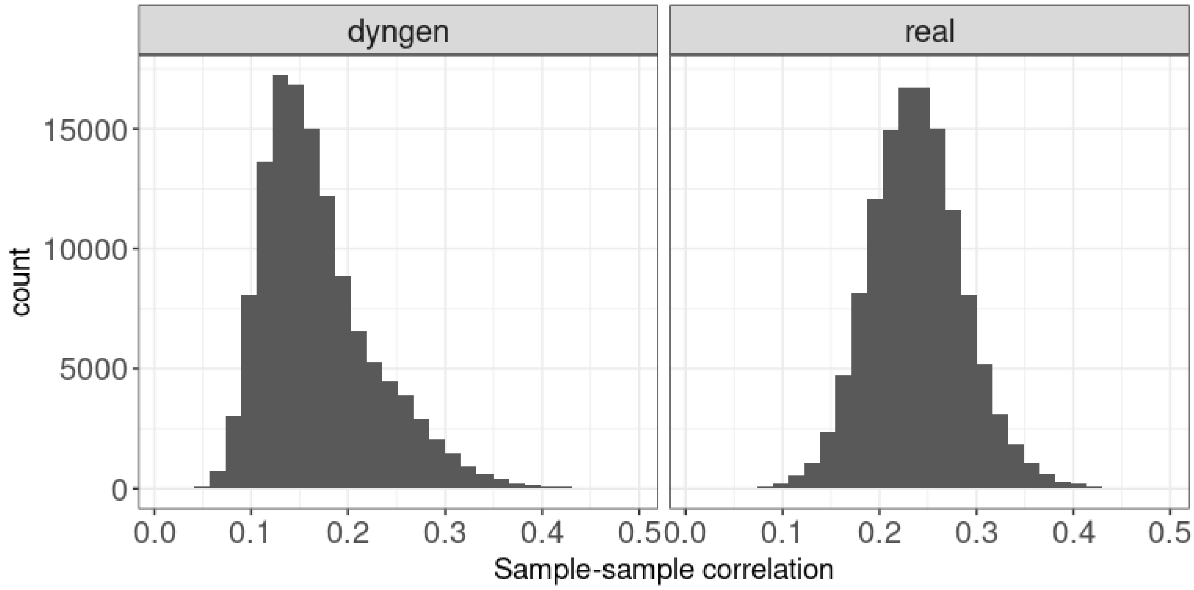

Feature-feature correlations

This plot illustrates the distribution of Spearman correlation coefficients for pairs of features, calculated from the log(CPM) values obtained via the `cpm` function from `edgeR`, with a prior.count of 2. Only non-constant features are considered.

```
ggplot(featureCorrDF, aes(x = Correlation)) + geom_histogram(bins = 30) +
  facet_wrap(~dataset, nrow = colRow[2]) +
  xlab("Feature-feature correlation") + thm
```

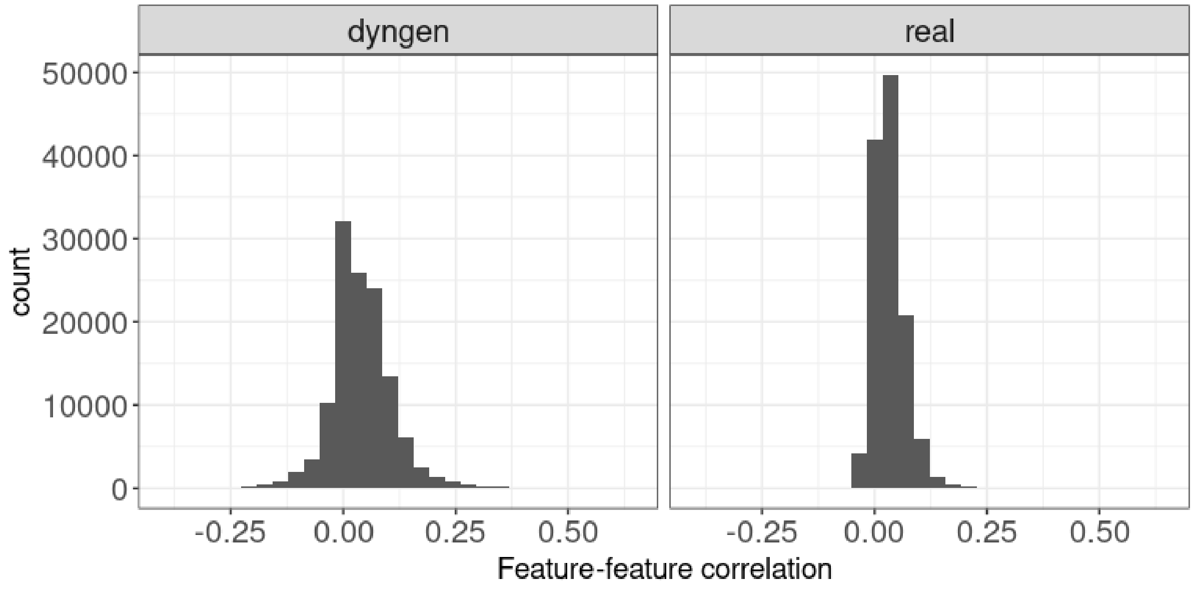

Library size vs fraction zeros

This scatter plot shows the association between the total count (column sums) and the fraction of zeros observed per sample.

```
ggplot(sampleDF, aes(x = Libsize, y = Fraczero)) +
  geom_point(size = 1, alpha = 0.5) +
  facet_wrap(~dataset, nrow = colRow[2]) +
  xlab("Library size") + ylab("Fraction zeros") + thm
```

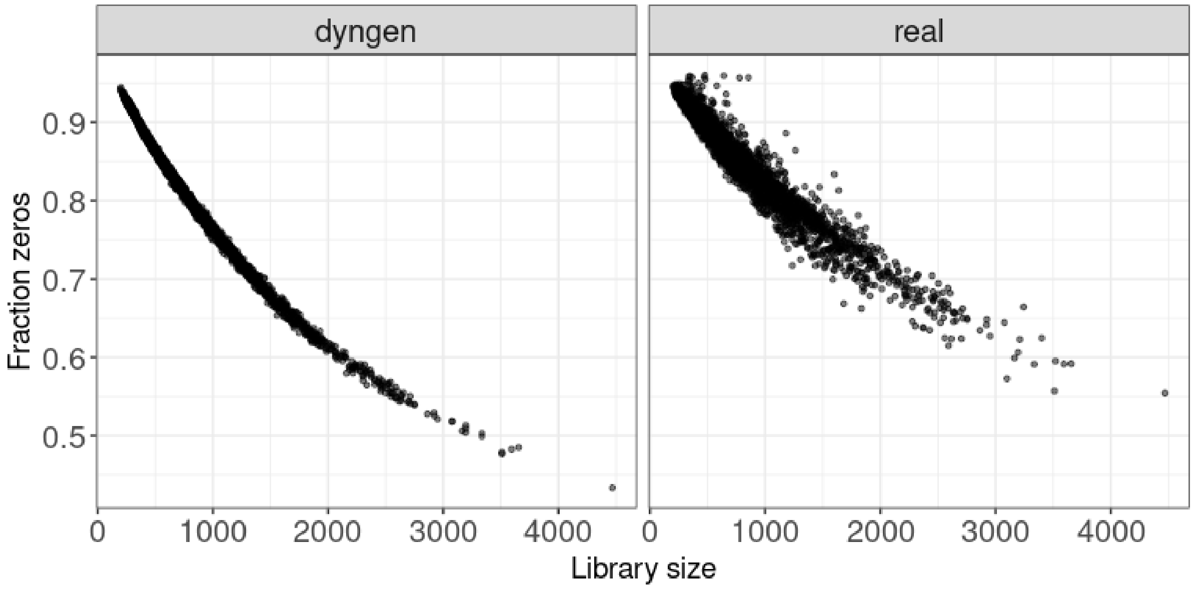

Mean expression vs fraction zeros

This scatter plot shows the association between the average abundance and the fraction of zeros observed per feature. The abundance is defined as the log(CPM) values as calculated by `edgeR`.

```
ggplot(featureDF, aes(x = AveLogCPM, y = Fraczero)) +
  geom_point(size = 0.75, alpha = 0.5) +
  facet_wrap(~dataset, nrow = colRow[2]) +
  xlab("Average log CPM") + ylab("Fraction zeros") + thm
```

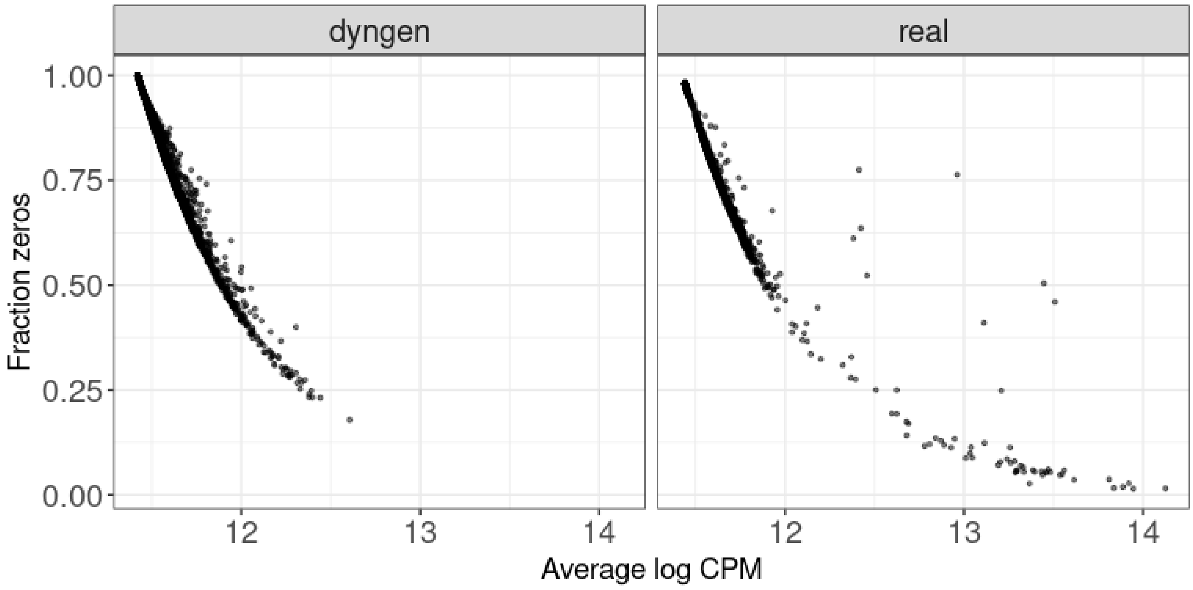

Supplementary Note 2: On scalability and runtime

In this vignette, we will take a look at the runtime of dyngen as the number of genes and the number of cells sampled is increased. We'll be using the bifurcating cycle backbone which is well known for its beautiful 3D butterfly shape!

```
library(dyngen)
library(tidyverse)

set.seed(1)

save_dir <- "scalability_and_runtime_runs"
if (!dir.exists(save_dir)) dir.create(save_dir, recursive = TRUE)

backbone <- backbone_bifurcating_cycle()
```

Initial run

We'll be running this simulation a few times, with different values for `num_cells` and `num_features` to assess the scalability of dyngen. An example of a resulting dyngen model is shown here.

```
num_cells <- 100
num_features <- 100
num_tfs <- nrow(backbone$module_info)
num_targets <- round((num_features - num_tfs) / 2)
num_hks <- num_features - num_targets - num_tfs

out <-
  initialise_model(
    backbone = backbone,
    num_tfs = num_tfs,
    num_targets = num_targets,
    num_hks = num_hks,
    num_cells = num_cells,
    gold_standard_params = gold_standard_default(
      census_interval = 1,
      tau = 100/3600
    ),
    simulation_params = simulation_default(
      census_interval = 10,
      ssa_algorithm = ssa_etl(tau = 300/3600),
      experiment_params = simulation_type_wild_type(
        num_simulations = num_cells / 10
      )
    ),
    verbose = FALSE
  ) %>%
  generate_dataset(make_plots = TRUE)
```

```
out$plot
```

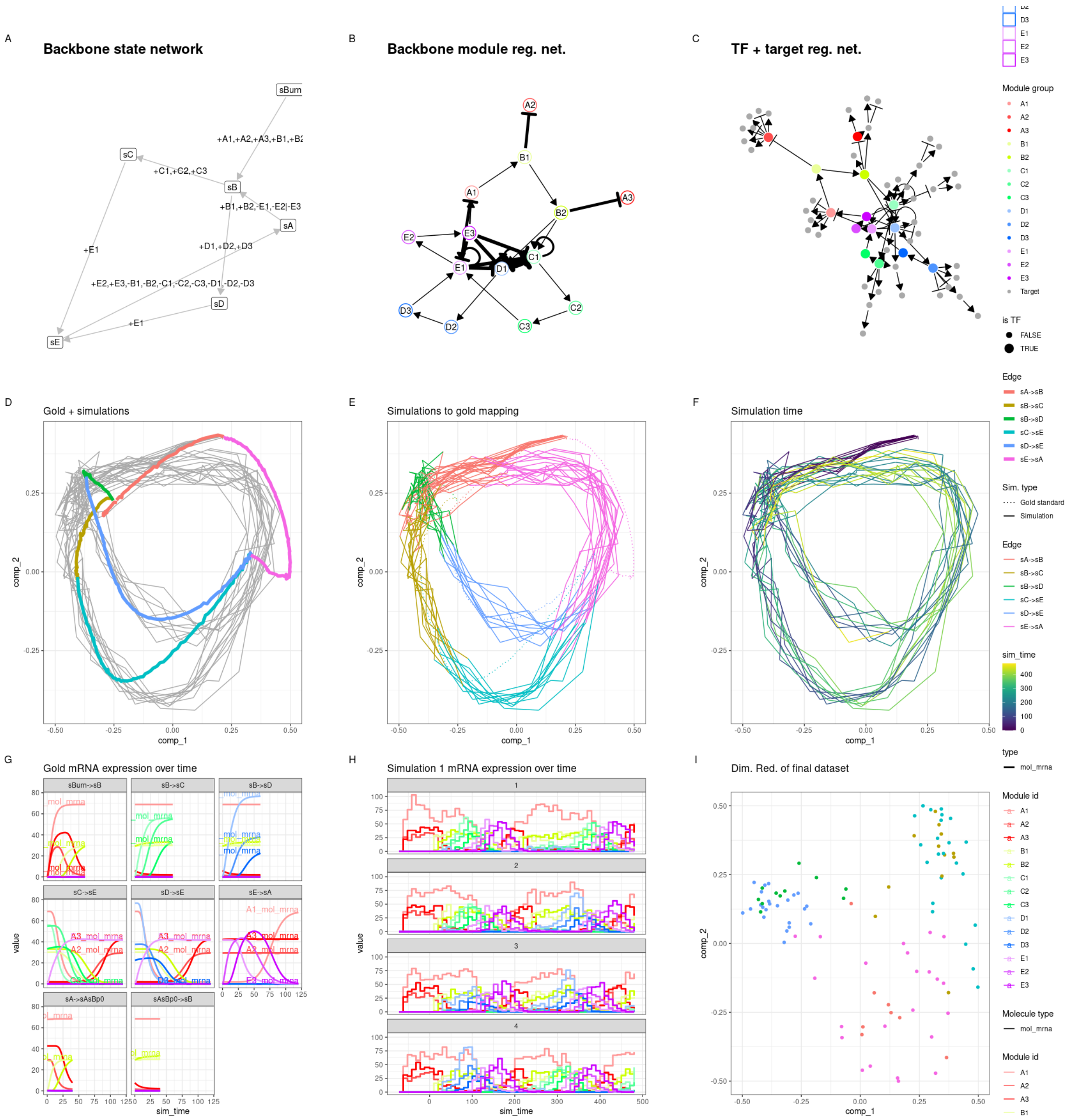

We tweaked some of the parameters by running this particular backbone once with `num_cells = 100` and `num_features = 100` and verifying that the new parameters still yield the desired outcome. The parameters we tweaked are:

- On average, 10 cells are sampled per simulation (e.g. `num_simulations = 100` and `num_cells = 1000`). You could increase this ratio to get a better cell count yield from a given set of simulations, but cells from the same simulation that are temporally close will have highly correlated expression profiles.
- Increased time steps `tau`. This will make the Gillespie algorithm slightly faster but might result in unexpected artifacts in the simulated data.
- `census_interval` increased from 4 to 10. This will cause dyngen to store an expression profile only every 10 time units. Since the total simulation time is xxx, each simulation will result in yyy data points. Note that on average only 10 data points are sampled per simulation.

For more information on parameter tuning, see the vignette 'Advanced: tuning the simulation parameters'.

Timing experiments

The simulations are run once with a large `num_features` and `num_cells`, a few times with varying `num_cells` and then once more with varying `num_features`. Every run is repeated three times in order

to get a bit more stable time measurements. Since some of the simulations can take over 10 minutes, the timings results of the simulations are cached in the 'scalability_and_runtime_runs' folder.'

```
settings <- bind_rows(
  tibble(num_cells = 10000, num_features = 10000, rep = 1), #, rep = seq_len(3)),
  crossing(
    num_cells = seq(1000, 10000, by = 1000),
    num_features = 100,
    rep = seq_len(3)
  ),
  crossing(
    num_cells = 100,
    num_features = seq(1000, 10000, by = 1000),
    rep = seq_len(3)
  )
) %>%
  mutate(filename = paste0(save_dir, "/cells", num_cells, "_feats", num_features,
    "_rep", rep, ".rds"))

timings <- pmap_dfr(settings, function(num_cells, num_features, rep, filename) {
  if (!file.exists(filename)) {
    set.seed(rep)

    cat("Running num_cells: ", num_cells, ", num_features: ", num_features,
      ", rep: ", rep, "\n", sep = "")
    num_tfs <- nrow(backbone$module_info)
    num_targets <- round((num_features - num_tfs) / 2)
    num_hks <- num_features - num_targets - num_tfs

    out <-
      initialise_model(
        backbone = backbone,
        num_tfs = num_tfs,
        num_targets = num_targets,
        num_hks = num_hks,
        num_cells = num_cells,
        gold_standard_params = gold_standard_default(
          census_interval = 1,
          tau = 100/3600
        ),
        simulation_params = simulation_default(
          census_interval = 10,
          ssa_algorithm = ssa_etl(tau = 300/3600),
          experiment_params = simulation_type_wild_type(
            num_simulations = num_cells / 10
          )
        ),
        verbose = FALSE
      ) %>%
      generate_dataset()

    tim <-
      get_timings(out$model) %>%
      mutate(rep, num_cells, num_features)

    write_rds(tim, filename, compress = "gz")
  }
```

```
  read_rds(filename)
})

timings_gr <-
  timings %>%
  group_by(group, task, num_cells, num_features) %>%
  summarise(time_elapsed = mean(time_elapsed), .groups = "drop")

timings_sum <-
  timings %>%
  group_by(num_cells, num_features, rep) %>%
  summarise(time_elapsed = sum(time_elapsed), .groups = "drop")
```

Simulate a large dataset (10k × 10k)

Below is shown the timings of each of the steps in simulating a dyngen dataset containing 10'000 genes and 10'000 features. The total simulation time required is 1147 seconds, most of which is spent performing the simulations itself.

```
timings0 <-
  timings_gr %>%
  filter(num_cells == 10000, num_features == 10000) %>%
  mutate(name = forcats::fct_rev(forcats::fct_inorder(paste0(group, ": ", task))))

ggplot(timings0) +
  geom_bar(aes(x = name, y = time_elapsed, fill = group), stat = "identity") +
  scale_fill_brewer(palette = "Dark2") +
  theme_classic() +
  theme(legend.position = "none") +
  coord_flip() +
  labs(x = NULL, y = "Time (s)", fill = "dyngen stage")
```

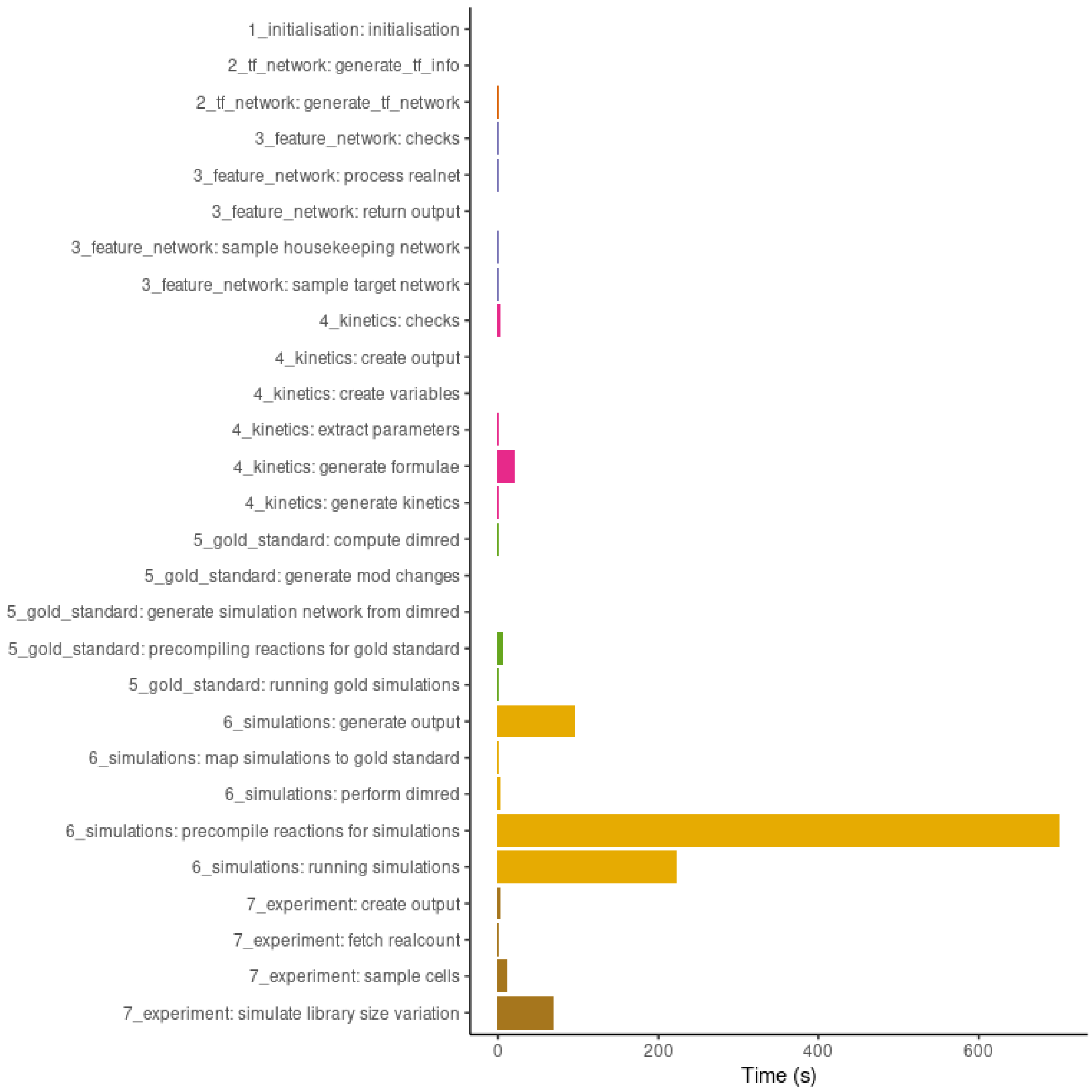

Increasing the number of cells

By increasing the number of cells from 1000 to 10'000 whilst keeping the number of features fixed, we can get an idea of how the simulation time scales w.r.t. the number of cells.

```
timings1 <-
  timings_gr %>%
  filter(num_features == 100) %>%
  group_by(num_cells, num_features, group) %>%
  summarise(time_elapsed = sum(time_elapsed), .groups = "drop")

ggplot(timings1) +
  geom_bar(aes(
    x = forcats::fct_inorder(as.character(num_cells)),
    y = time_elapsed,
    fill = forcats::fct_inorder(group)),
    stat = "identity") +
  theme_classic() +
  scale_fill_brewer(palette = "Dark2") +
  labs(x = "Number of cells", y = "Average time (s)", fill = "dyngen step")
```

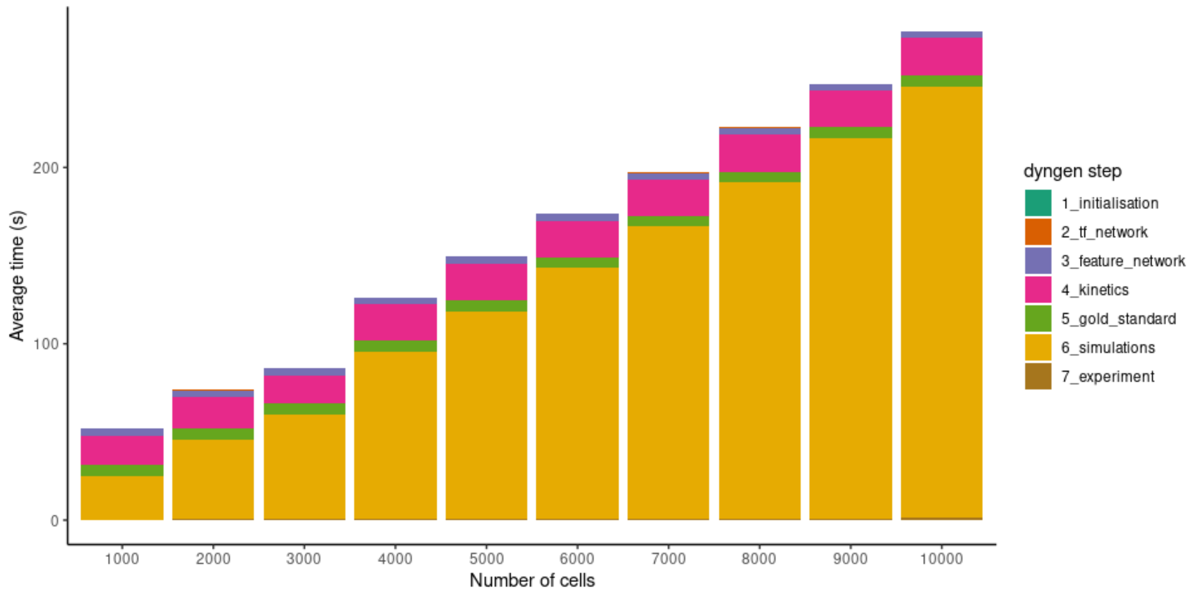

It seems the execution time scales linearly w.r.t. the number of cells. This makes sense, because as the number of cells are increased, so do we increase the number of simulations made (which is not necessarily mandatory). Since the simulations are independent of each other and take up the most time, the execution time will scale linearly.

```
ggplot(timings_sum %>% filter(num_features == 100)) +
  theme_bw() +
  geom_point(aes(num_cells, time_elapsed)) +
  scale_x_continuous(limits = c(0, 10000)) +
  scale_y_continuous(limits = c(0, 300)) +
  geom_abline(intercept = 22.097, slope = 0.0252) +
  labs(x = "Number of cells", y = "Execution time (s)")
```

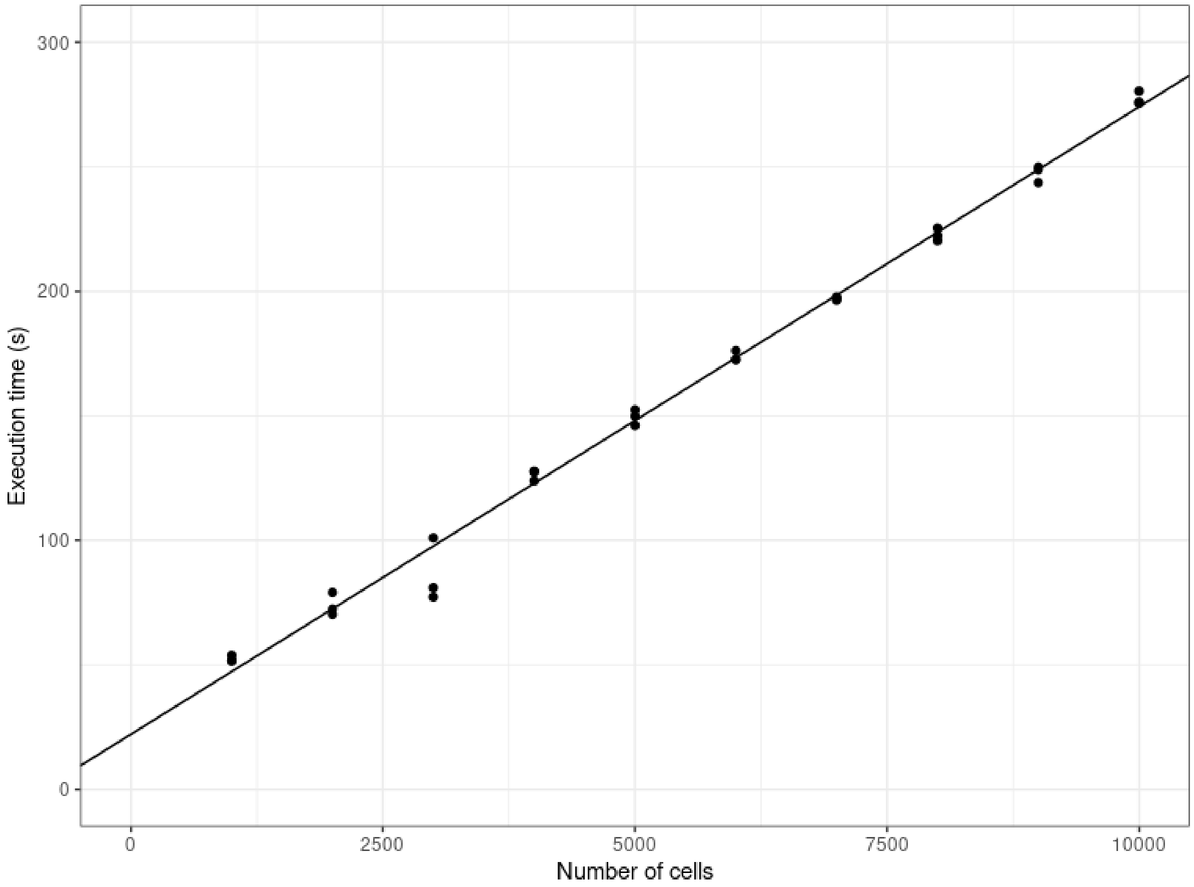

Increasing the number of features

By increasing the number of features from 1000 to 10’000 whilst keeping the number of cells fixed, we can get an idea of how the simulation time scales w.r.t. the number of features

```
timings2 <-
  timings_gr %>%
  filter(num_cells == 100) %>%
  group_by(num_cells, num_features, group) %>%
  summarise(time_elapsed = sum(time_elapsed), .groups = "drop")

ggplot(timings2) +
  geom_bar(aes(
    x = forcats::fct_inorder(as.character(num_features)),
    y = time_elapsed,
    fill = forcats::fct_inorder(group)),
    stat = "identity") +
  theme_classic() +
  scale_fill_brewer(palette = "Dark2") +
  labs(x = "Number of features", y = "Average time (s)", fill = "dyngen step")
```

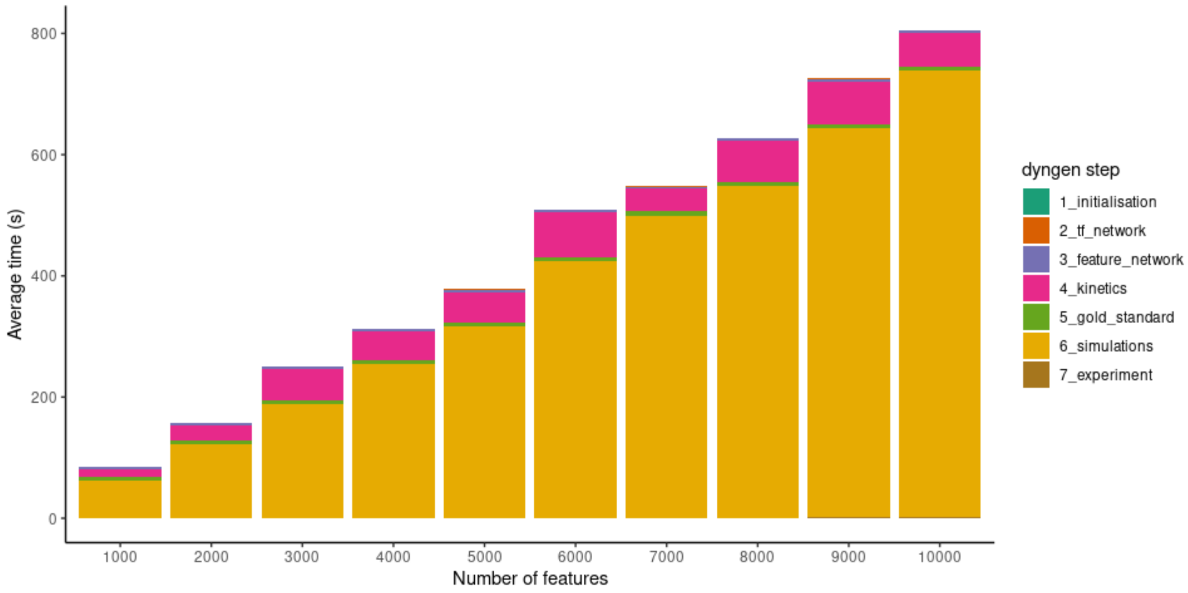

It seems the execution time also scales linearly w.r.t. the number of features. As more genes are added to the underlying gene regulatory network, the density of the graph doesn't change, so it makes sense that the execution time also scales linearly w.r.t. the number of features.

```
ggplot(timings_sum %>% filter(num_cells == 100)) +
  theme_bw() +
  geom_point(aes(num_features, time_elapsed)) +
  scale_x_continuous(limits = c(0, 10000)) +
  scale_y_continuous(limits = c(0, 850)) +
  geom_abline(intercept = 0.5481, slope = 0.07988) +
  labs(x = "Number of features", y = "Execution time (s)")
```

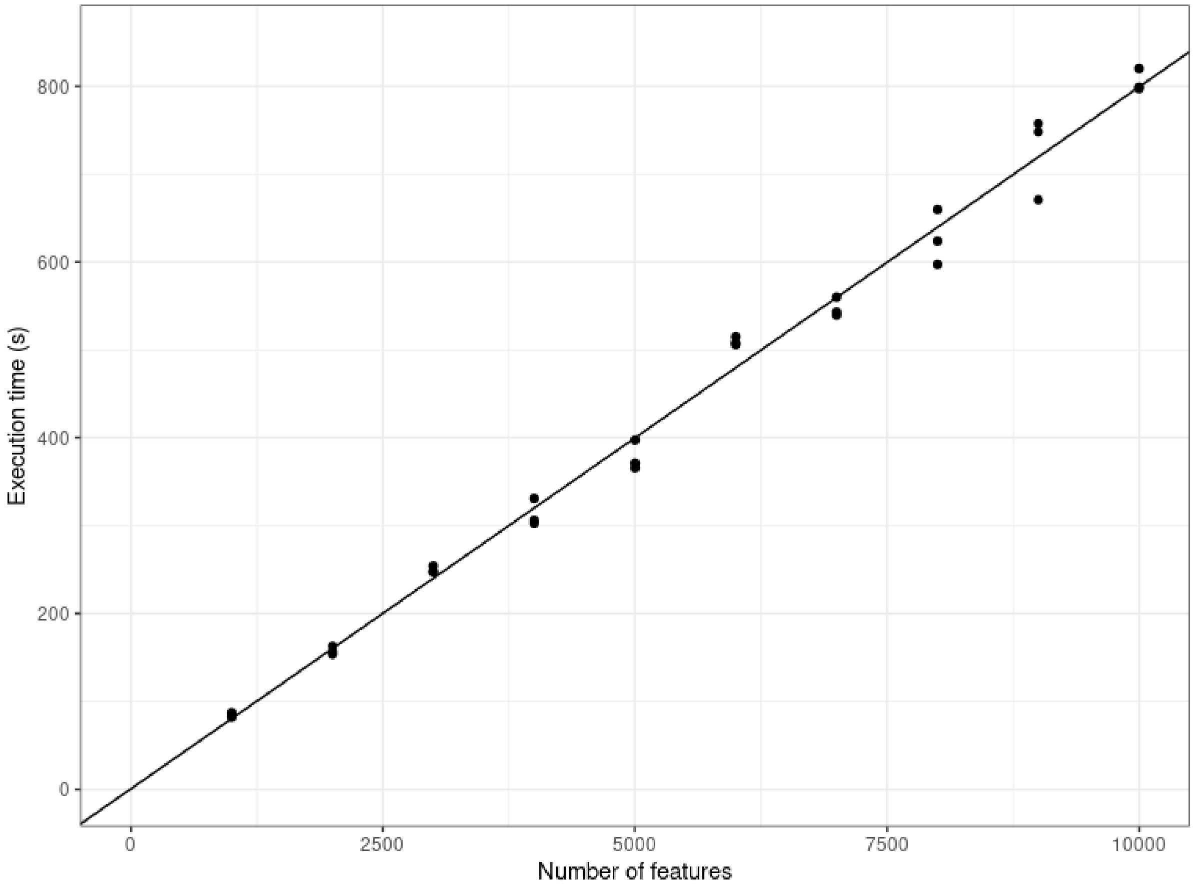

Execution platform

These timings were measured using 30 (out of 32) threads using a AMD Ryzen 9 5950X clocked at 3.4GHz.

Supplementary References

[1] Koen Van den Berge et al. "Trajectory-Based Differential Expression Analysis for Single-Cell Sequencing Data". In: *Nature Communications* 11.1 (Mar. 2020), p. 1201. ISSN: 2041-1723. DOI: 10.1038/s41467-020-14766-3.

[2] Charlotte Soneson and Mark D. Robinson. "Towards Unified Quality Verification of Synthetic Count Data with countsimQC". en. In: *Bioinformatics* 34.4 (Feb. 2018), pp. 691–692. ISSN: 1367-4803. DOI: 10.1093/bioinformatics/btx631.

[3] Mark D. Robinson, Davis J. McCarthy, and Gordon K. Smyth. "edgeR: A Bioconductor Package for Differential Expression Analysis of Digital Gene Expression Data". In: *Bioinformatics* 26.1 (Jan. 2010), pp. 139–140. ISSN: 1367-4803. DOI: 10.1093/bioinformatics/btp616.

[4] Michael I. Love, Wolfgang Huber, and Simon Anders. "Moderated Estimation of Fold Change and Dispersion for RNA-Seq Data with DESeq2". In: *Genome Biology* 15.12 (Dec. 2014), p. 550. ISSN: 1474-760X. DOI: 10.1186/s13059-014-0550-8.

[5] Yunshun Chen, Aaron T. L. Lun, and Gordon K. Smyth. "Differential Expression Analysis of Complex RNA-Seq Experiments Using edgeR". In: *Statistical Analysis of Next Generation Sequencing Data*. Ed. by Somnath Datta and Dan Nettleton. Cham: Springer International Publishing, 2014, pp. 51–74. ISBN: 978-3-319-07212-8. DOI: 10.1007/978-3-319-07212-8_3.

[6] Mark D. Robinson and Alicia Oshlack. "A Scaling Normalization Method for Differential Expression Analysis of RNA-Seq Data". In: *Genome Biology* 11.3 (Mar. 2010), R25. ISSN: 1474-760X. DOI: 10.1186/gb-2010-11-3-r25.
