# Supplementary material for: Spearheading future omics analyses using dyngen, a multi-modal simulator of single cells
Source: Nat Commun. 2021 Jun 24;12:3942. doi: 10.1038/s41467-021-24152-2 (PMC8225657; doi:10.1038/s41467-021-24152-2)
Supplement: Supplementary file 3 — Reporting Summary [file 41467_2021_24152_MOESM3_ESM.pdf]

nature research

Corresponding author(s): Yvan Saeys

Last updated by author(s): May 12, 2021

Reporting Summary

Nature Research wishes to improve the reproducibility of the work that we publish. This form provides structure for consistency and transparency in reporting. For further information on Nature Research policies, see our Editorial Policies and the Editorial Policy Checklist.

Statistics

For all statistical analyses, confirm that the following items are present in the figure legend, table legend, main text, or Methods section.

n/a	Confirmed	
☐	☒	The exact sample size (*n*) for each experimental group/condition, given as a discrete number and unit of measurement
☐	☒	A statement on whether measurements were taken from distinct samples or whether the same sample was measured repeatedly
☐	☒	The statistical test(s) used AND whether they are one- or two-sided<br>*Only common tests should be described solely by name; describe more complex techniques in the Methods section.*
☐	☒	A description of all covariates tested
☐	☒	A description of any assumptions or corrections, such as tests of normality and adjustment for multiple comparisons
☒	☐	A full description of the statistical parameters including central tendency (e.g. means) or other basic estimates (e.g. regression coefficient) AND variation (e.g. standard deviation) or associated estimates of uncertainty (e.g. confidence intervals)
☐	☒	For null hypothesis testing, the test statistic (e.g. *F*, *t*, *r*) with confidence intervals, effect sizes, degrees of freedom and *P* value noted<br>*Give P values as exact values whenever suitable.*
☒	☐	For Bayesian analysis, information on the choice of priors and Markov chain Monte Carlo settings
☒	☐	For hierarchical and complex designs, identification of the appropriate level for tests and full reporting of outcomes
☒	☐	Estimates of effect sizes (e.g. Cohen's *d*, Pearson's *r*), indicating how they were calculated

Our web collection on statistics for biologists contains articles on many of the points above.

Software and code

Policy information about availability of computer code

Data collection

All data was generated with R 4.0.3 and dyngen 1.0.0. dyngen is available as an open-source R package on CRAN at https://cran.r-project.org/package=dyngen. Downstream dependencies:
* anndata 0.7.5.1
* assertthat 0.2.1
* bit 4.0.4
* bit64 4.0.5
* codetools 0.2-18
* colorspace 2.0-0
* compiler 4.0.4
* crayon 1.4.1.9000
* data.table 1.13.4
* DBI 1.1.1
* debugme 1.1.0
* desc 1.2.0
* digest 0.6.27
* dplyr 1.0.5
* dynutils 1.0.6
* ellipsis 0.3.1
* fansi 0.4.2
* farver 2.1.0
* future 1.20.1
* future.apply 1.7.0
* generics 0.1.0
* ggforce 0.3.2

* ggplot2 3.3.3
* ggraph 2.0.4
* ggrepel 0.9.0
* GillespieSSA2 0.2.7
* globals 0.14.0
* glue 1.4.2
* graphlayouts 0.7.1
* grid 4.0.4
* gridExtra 2.3
* gtable 0.3.0
* hdf5r 1.3.3
* hms 1.0.0
* igraph 1.2.6
* irlba 2.3.3
* jsonlite 1.7.2
* lattice 0.20-41
* lifecycle 1.0.0
* lisi 1.0
* listenv 0.8.0
* lmds 0.1.0
* magrittr 2.0.1
* MASS 7.3-53
* Matrix 1.3-2
* matrixStats 0.57.0
* munsell 0.5.0
* parallel 4.0.4
* parallelly 1.21.0
* patchwork 1.1.1
* pbapply 1.4-3
* pillar 1.5.1
* pkgconfig 2.0.3
* plyr 1.8.6
* polyclip 1.10-0
* proxyC 0.1.5
* purrr 0.3.4
* R6 2.5.0
* RANN 2.6.1
* Rcpp 1.0.6
* RcppParallel 5.0.2
* RcppXPtrUtils 0.1.1
* readr 1.4.0
* remotes 2.2.0
* reshape2 1.4.4
* reticulate 1.18-9007
* rlang 0.4.10
* rprojroot 2.0.2
* scales 1.1.1
* sctransform 0.3.2
* stringi 1.5.3
* stringr 1.4.0
* tibble 3.0.5
* tidygraph 1.2.0
* tidyr 1.1.2
* tidyselect 1.1.0
* tools 4.0.4
* tweenr 1.0.1
* utf8 1.1.4
* vctrs 0.3.6
* viridis 0.5.1
* viridisLite 0.3.0

Data analysis

Data was analysed with code available at https://github.com/dynverse/dyngen_manuscript. Downstream dependencies:
* anndata 0.7.5.1
* assertthat 0.2.1
* babelwhale 1.0.1
* bit 4.0.4
* bit64 4.0.5
* carrier 0.1.0
* cellAlign 0.1.0
* codetools 0.2-18
* colorspace 2.0-0
* compiler 4.0.4
* crayon 1.4.1.9000
* data.table 1.13.4
* DBI 1.1.1
* debugme 1.1.0

* desc 1.2.0
* digest 0.6.27
* dplyr 1.0.5
* dtw 1.22-3
* dynparam 1.0.1
* dynutils 1.0.6
* dynwrap 1.2.2
* ellipsis 0.3.1
* fansi 0.4.2
* farver 2.1.0
* future 1.20.1
* future.apply 1.7.0
* generics 0.1.0
* ggforce 0.3.2
* ggplot2 3.3.3
* ggraph 2.0.4
* ggrepel 0.9.0
* GillespieSSA2 0.2.7
* globals 0.14.0
* glue 1.4.2
* graphlayouts 0.7.1
* grid 4.0.4
* gridExtra 2.3
* gtable 0.3.0
* gtools 3.8.2
* hdf5r 1.3.3
* hms 1.0.0
* igraph 1.2.6
* irlba 2.3.3
* jsonlite 1.7.2
* lattice 0.20-41
* lifecycle 1.0.0
* lisi 1.0
* listenv 0.8.0
* lmds 0.1.0
* magrittr 2.0.1
* MASS 7.3-53
* Matrix 1.3-2
* matrixStats 0.57.0
* munsell 0.5.0
* parallel 4.0.4
* parallelly 1.21.0
* patchwork 1.1.1
* pbapply 1.4-3
* pheatmap 1.0.12
* pillar 1.5.1
* pkgconfig 2.0.3
* plyr 1.8.6
* polyclip 1.10-0
* pracma 2.2.9
* processx 3.4.5
* proxy 0.4-24
* proxyC 0.1.5
* ps 1.6.0
* purrr 0.3.4
* R6 2.5.0
* RANN 2.6.1
* rappdirs 0.3.3
* RColorBrewer 1.1-2
* Rcpp 1.0.6
* RcppParallel 5.0.2
* RcppXPtrUtils 0.1.1
* readr 1.4.0
* remotes 2.2.0
* reshape2 1.4.4
* reticulate 1.18-9007
* rlang 0.4.10
* rprojroot 2.0.2
* scales 1.1.1
* sctransform 0.3.2
* scvelo 0.1.0.9000
* stringi 1.5.3
* stringr 1.4.0
* tibble 3.0.5
* tidygraph 1.2.0
* tidyr 1.1.2

* tidyselect 1.1.0
* tools 4.0.4
* tweenr 1.0.1
* utf8 1.1.4
* vctrs 0.3.6
* viridis 0.5.1
* viridisLite 0.3.0
* yaml 2.2.1

For manuscripts utilizing custom algorithms or software that are central to the research but not yet described in published literature, software must be made available to editors and reviewers. We strongly encourage code deposition in a community repository (e.g. GitHub). See the Nature Research guidelines for submitting code & software for further information.

Data

Policy information about availability of data

All manuscripts must include a data availability statement. This statement should provide the following information, where applicable:
- Accession codes, unique identifiers, or web links for publicly available datasets
- A list of figures that have associated raw data
- A description of any restrictions on data availability

Source data for Figure 2A and Supp. Fig. 1E are available in "Source Data.xlsx" in the sheet named "trajectory_alignment_summary"
Source data for Figure 2B and Supp. Fig. 2D are available in "Source Data.xlsx" in the sheet named "RNA_velocity_summary"
Source data for Figure 2C and Supp. Fig. 3B are available in "Source Data.xlsx" in the sheet named "CSNI_summary"

All code and data required to reproduce the analysis are available on GitHub at https://github.com/dynverse/dyngen_manuscript. The datasets generated for the different use cases are available on Zenodo with record number (doi: 10.5281/zenodo.4637926).

Field-specific reporting

Please select the one below that is the best fit for your research. If you are not sure, read the appropriate sections before making your selection.

☒ Life sciences ☐ Behavioural & social sciences ☐ Ecological, evolutionary & environmental sciences

For a reference copy of the document with all sections, see nature.com/documents/nr-reporting-summary-flat.pdf

Life sciences study design

All studies must disclose on these points even when the disclosure is negative.

Sample size	Each use-case experiment consists of 40 or 42 datasets and is based on the number of different dataset generators available for each task. As it is an in silico benchmark, this could have been scaled up to an arbitrary number.
Data exclusions	No data was excluded from this study.
Replication	Each unique backbone was simulated by dyngen with at least three different seeds. As the methods used in this manuscript are inherently stochastic, they will produce slightly different results between runs, as expected.
Randomization	Randomization is not relevant for this study as no human participation is involved in any of the in silico experiments.
Blinding	Blinding is not relevant for this study as no human participation is involved in any of the in silico experiments.

Reporting for specific materials, systems and methods

We require information from authors about some types of materials, experimental systems and methods used in many studies. Here, indicate whether each material, system or method listed is relevant to your study. If you are not sure if a list item applies to your research, read the appropriate section before selecting a response.

Materials & experimental systems

n/a	Involved in the study	
☒	☐	Antibodies
☒	☐	Eukaryotic cell lines
☒	☐	Palaeontology and archaeology
☒	☐	Animals and other organisms
☒	☐	Human research participants
☒	☐	Clinical data
☒	☐	Dual use research of concern

Methods

n/a	Involved in the study	
☒	☐	ChIP-seq
☒	☐	Flow cytometry
☒	☐	MRI-based neuroimaging
